# Loss of the Inducible Hsp70 Delays the Inflammatory Response to Skeletal Muscle Injury and Severely Impairs Muscle Regeneration

**DOI:** 10.1371/journal.pone.0062687

**Published:** 2013-04-23

**Authors:** Sarah M. Senf, Travis M. Howard, Bumsoo Ahn, Leonardo F. Ferreira, Andrew R. Judge

**Affiliations:** 1 Department of Physical Therapy, University of Florida, Gainesville, Florida, United States of America; 2 Department of Applied Physiology and Kinesiology, University of Florida, Gainesville, Florida, United States of America; California State University Fullerton, United States of America

## Abstract

Skeletal muscle regeneration following injury is a highly coordinated process that involves transient muscle inflammation, removal of necrotic cellular debris and subsequent replacement of damaged myofibers through secondary myogenesis. However, the molecular mechanisms which coordinate these events are only beginning to be defined. In the current study we demonstrate that Heat shock protein 70 (Hsp70) is increased following muscle injury, and is necessary for the normal sequence of events following severe injury induced by cardiotoxin, and physiological injury induced by modified muscle use. Indeed, Hsp70 ablated mice showed a significantly delayed inflammatory response to muscle injury induced by cardiotoxin, with nearly undetected levels of both neutrophil and macrophage markers 24 hours post-injury. At later time points, Hsp70 ablated mice showed sustained muscle inflammation and necrosis, calcium deposition and impaired fiber regeneration that persisted several weeks post-injury. Through rescue experiments reintroducing Hsp70 intracellular expression plasmids into muscles of Hsp70 ablated mice either prior to injury or post-injury, we confirm that Hsp70 optimally promotes muscle regeneration when expressed during both the inflammatory phase that predominates in the first four days following severe injury and the regenerative phase that predominates thereafter. Additional rescue experiments reintroducing Hsp70 protein into the extracellular microenvironment of injured muscles at the onset of injury provides further evidence that Hsp70 released from damaged muscle may drive the early inflammatory response to injury. Importantly, following induction of physiological injury through muscle reloading following a period of muscle disuse, reduced inflammation in 3-day reloaded muscles of Hsp70 ablated mice was associated with preservation of myofibers, and increased muscle force production at later time points compared to WT. Collectively our findings indicate that depending on the nature and severity of muscle injury, therapeutics which differentially target both intracellular and extracellular localized Hsp70 may optimally preserve muscle tissue and promote muscle functional recovery.

## Introduction

Heat shock protein 70 (Hsp70) expression is induced in skeletal muscle in response to a variety of physiological insults [1], [2], and plays a significant role in protecting against cellular damage and dysfunction. The mechanistic nature of Hsp70-mediated cellular protection is believed to be related to both its role as a protein chaperone in regulating protein quality [1], [3], [4] and its ability to regulate the activities of various signaling proteins and transcription factors, including jun N-terminal kinase (JNK) [5], p38 mitogen-activated protein kinase [6], nuclear factor κB (NF-κB) [5], [6], [7], [8] and forkhead boxO (FoxO) [8], [9]. Due to the significance of protein quality control and these signaling proteins in regulating skeletal muscle mass, regeneration and function [10], [11], [12], [13], [14], [15], therapies targeting Hsp70 intracellular induction in skeletal muscle have proved beneficial in animal models of muscle atrophy [8] muscle damage [16], [17], aging [18] and recently, muscular dystrophy [19].

In addition to the several intracellular functions of Hsp70, there is also evidence to support distinct functions of extracellular localized Hsp70 (eHsp70) [20]. A handful of studies now indicate that Hsp70 is released from cells following stress or injury [21], [22], [23], [24], [25], [26], and that this plays an important role in activating the innate immune response [27], [28], [29]. In skeletal muscle, the release of intracellular proteins in the event of muscle fiber injury plays a critical role in immune cell activation and recruitment to the site of muscle injury, which subsequently regulates muscle regeneration. However, the specific muscle proteins involved in this process are only beginning to be understood. Since in vitro studies indicate that Hsp70 can bind to and activate both neutrophils [30] and macrophages [29], two inflammatory cell populations which infiltrate damaged muscle and modulate regeneration following injury, Hsp70 derived from injured muscle is an excellent candidate in the regulation of the skeletal muscle inflammatory response to injury. However, despite this evidence, to our knowledge, no studies have (a) determined whether Hsp70 regulates the inflammatory response following to muscle injury or, (b) determined whether Hsp70 is required for normal muscle regeneration. In fact, to our knowledge, no studies currently exist demonstrating the requirement for Hsp70 in any form of skeletal muscle plasticity.

Identifying proteins which regulate the inflammatory response to skeletal muscle injury is critical to our understanding of skeletal muscle regeneration as it relates to both acute muscle injury and the progressive myopathies that are associated with chronic bouts of muscle damage and sustained muscle inflammation. Infiltration of immune cells following acute muscle injury is necessary for normal regeneration and recovery, which is related to their role in the phagocytic removal of damaged cellular debris and the secretion of factors which promote muscle regeneration [31], [32], [33]. Paradoxically, inflammatory cell infiltration into muscle can also amplify muscle damage [34], [35] and inhibit regenerative processes [10]. Furthermore, during conditions associated with chronic bouts of muscle injury, including muscular dystrophy [36], [37] and hind limb ischemia associated with vascular disease [38], recurring muscle inflammation is believed to be a central contributor to the skeletal myopathy that accompanies these diseases. Therefore, identifying novel proteins which regulate the inflammatory response to muscle injury as it relates to regeneration could open up new therapeutic avenues to both enhance muscle recovery following acute injuries and delay the progression of skeletal myopathy associated with several diseases.

The current study utilized *WT* and *Hsp70* ^−/−^ mice to determine whether Hsp70 is required for normal muscle regeneration following acute muscle injury and whether this is mediated through its regulation of the skeletal muscle inflammatory response. We demonstrate that Hsp70^−/−^ mice have a significantly delayed muscle inflammatory response as early as 24 hours post muscle-injury, which was associated with severely impaired muscle regeneration and recovery at later time points. Rescue experiments reintroducing Hsp70 specifically into skeletal muscle either prior to injury or post-injury provide further evidence that skeletal muscle-derived Hsp70 is indeed important for muscle regeneration through regulating both the early inflammatory and regenerative phases following muscle injury.

## Results

### Genetic ablation of Hsp70 alters skeletal muscle fiber size

**Figure 1:**
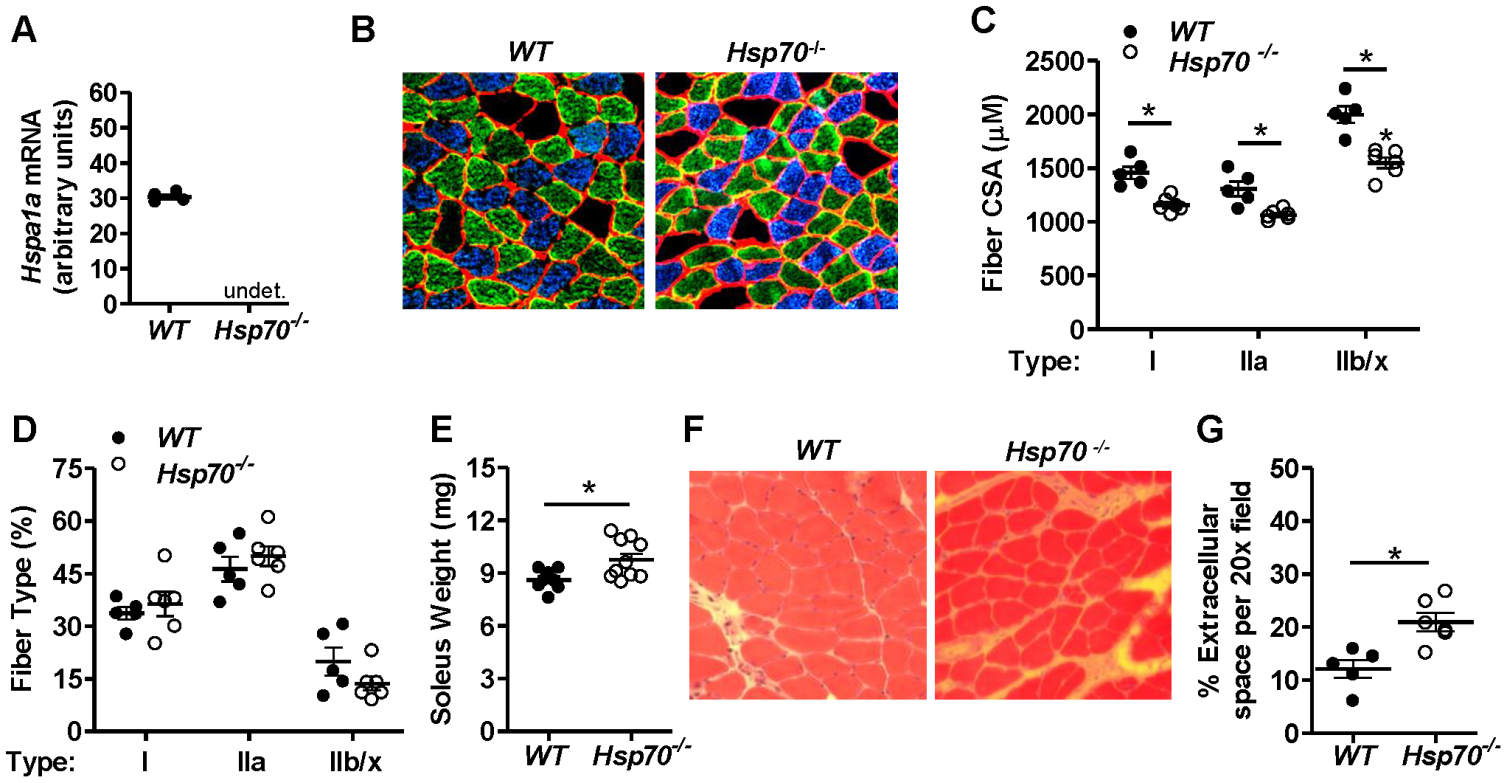
Hsp70 genetic ablation alters skeletal muscle fiber size and quality. (A) Genetic deletion of *Hspa1a* which codes for the inducible 70 kDa Hsp was confirmed in *Hsp70* ^−/−^ mice via qRT-PCR using primers for the *Hspa1a* gene and cDNA generated from gastrocnemius muscles from *WT* and *Hsp70* ^−/−^ mice, n = 4 mice/group. (B–D) Soleus muscle cross-sections from 12-week old *WT* and *Hsp70* ^−/−^ mice were incubated with antibodies for laminin, to outline muscle fiber membranes, and antibodies for Type I and Type IIa myosin heavy chain to distinguish Type I (blue), Type IIa (green) and Type IIb/x (black) muscle fibers (B). The average CSA of each fiber type (C) and the fiber type distribution (D) were calculated from entire muscle cross-sections, n =  at least 5 mice/group. E) Average soleus muscle mass from 12 week-old *WT* and *Hsp70* ^−/−^ mice, n =  at least 4 mice (8 muscles) per group (F & G). Soleus muscle cross-sections from *WT* and *Hsp70* ^−/−^ mice were H&E-stained for morphological assessment (F) and the average extracellular space surrounding myofibers was calculated, n =  at least 5 mice/group (G). All data represent mean ± SEM, *P<0.05.

The primary interest of the current study was in determining the requirement of Hsp70 for the acute skeletal muscle injury response in post-natal skeletal muscle. However, to our knowledge, the skeletal muscle phenotype of mice which lack Hsp70 during embryonic growth and post-natal development has not been determined. Therefore, the use of lifelong *Hsp70* ^−/−^ mice in the current study also allowed us to determine whether lifelong knockout of Hsp70 results in an overt skeletal muscle phenotype. Confirmation that *Hsp70* ^−/−^ mice do not express *Hsp70* was verified through qRT-PCR on gastrocnemius muscles from *WT* and *Hsp70* ^−/−^ mice using primers for *Hspa1a*, which codes for Hsp70 and is shown in Figure 1A. We next determined whether skeletal muscle fiber cross-sectional area (CSA), mass and morphology were altered in adult (12-week old) *Hsp70* ^−/−^ mice compared to *WT* mice. To more specifically determine the effect of Hsp70 knockout on the fiber CSA of each muscle fiber type, we performed immunohistochemistry on soleus muscle cross-sections to identify Type I, Type IIa and Type IIb/x muscle fibers (Figure 1B), and calculated the average CSA for each fiber type across entire muscle cross-sections. *WT* mice had an average CSA of 1457 µm^2^ for Type I fibers, 1307 µm^2^ for Type IIa fibers and 2000 µm^2^ for Type IIb/x fibers (Figure 1C). Compared to *WT* mice, *Hsp70* ^−/−^ mice had significant deficits in fiber CSA across all muscle fiber types, with an average CSA of 1157 µm^2^ for Type I fibers (21% smaller than *WT*), 1063 µm^2^ for Type IIa fibers (19% smaller than *WT*) and 1549 µm^2^ for Type IIb/x fibers (23% smaller than *WT*). No differences were seen in muscle fiber type composition between muscles of *WT* and *Hsp70* ^−/−^ mice (Figure 1D). Interestingly however, despite the smaller cross-sectional area of muscle fibers in soleus muscles from *Hsp70* ^−/−^ mice compared to *WT*, the average weight of soleus muscles from *Hsp70* ^−/−^ mice was significantly greater than *WT* (*Hsp70* ^−/−^  = 9.7 mg, *WT*  = 8.6 mg). One potential explanation for this finding may be a difference in soleus muscle quality between *WT* and *Hsp70* ^−/−^ mice (i.e. the amount of non-muscle fiber tissue in the muscle). We therefore grossly assessed the quality of soleus muscles from *WT* and *Hsp70* ^−/−^ mice at this time point through H&E-staining of muscle cross-sections (Figure 1F). Quantification of the extracellular tissue in entire muscle cross-sections demonstrated that *Hsp70* ^−/−^ mice indeed have increased extracellular tissue surrounding myofibers compared to *WT* (Figure 1G), which indicates that muscles from *Hsp70* ^−/−^ mice not only have smaller myofibers but may also have decreased muscle quality.

### Hsp70^−/−^ mice show impaired muscle regeneration and recovery following injury

**Figure 2:**
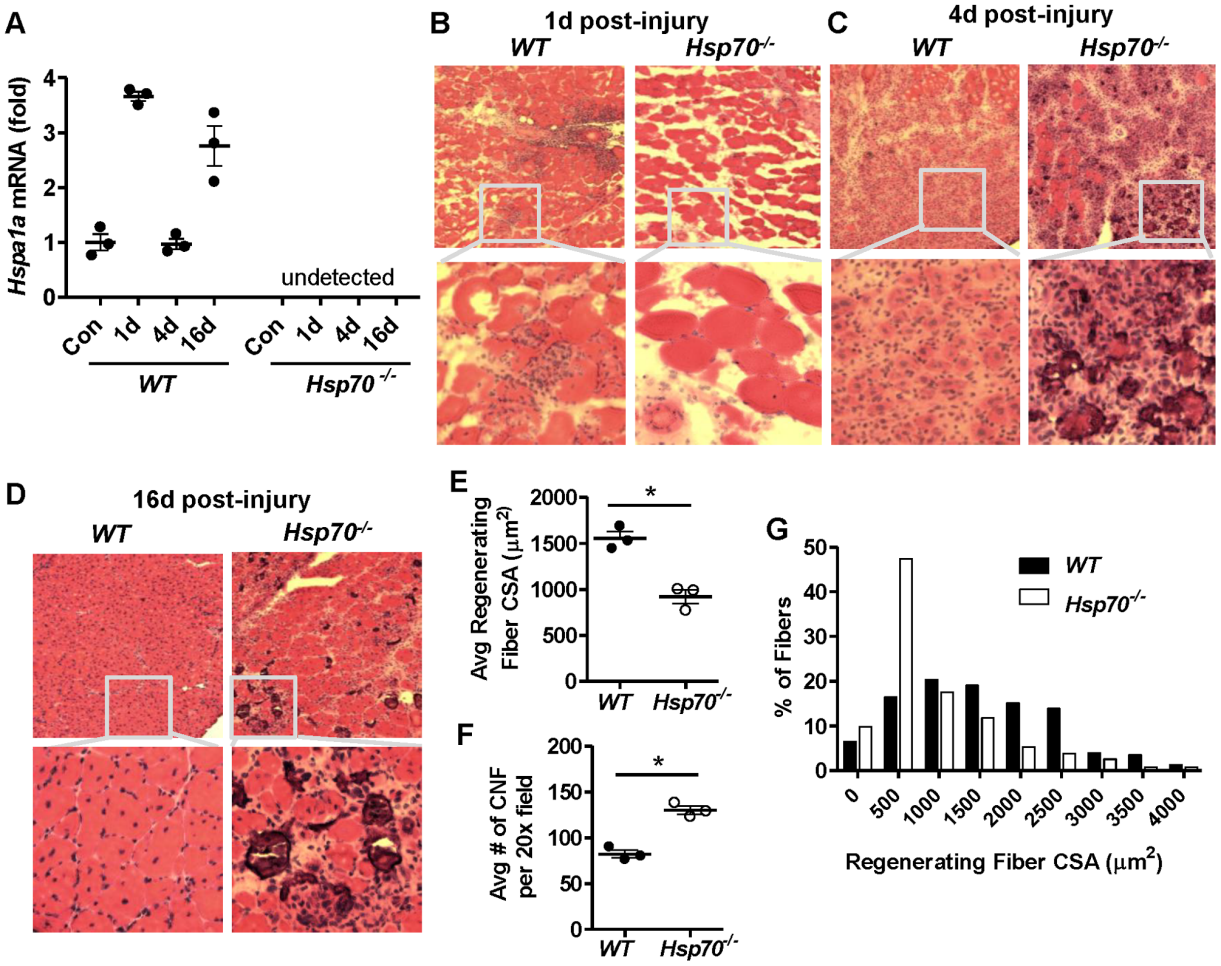
Injured TA muscles from *Hsp70* ^−/−^ mice show impaired muscle regeneration and recovery. (A) *Hsp70* (*Hspa1a*) mRNA was measured in control and injured muscles from *WT* and *Hsp70* ^−/−^ mice 1 day, 4 days and 16 days post cardiotoxin-injury. Morphological assessment of injured muscles from *WT* and *Hsp70* ^−/−^ mice was performed via H&E staining of muscle cross-sections 1 day (B), 4 days (C) or 16 days (D) post-injury. (E–G) Muscle regeneration was assessed in muscles from *WT* and *Hsp70* ^−/−^ mice 16 days post-injury via quantification of the average CSA (E) and number (F) of regenerating fibers containing centralized nuclei (CNF), which is further represented in a fiber size frequency distribution (G). All data represent mean ± SEM, and all data are representative of at least n = 3 mice per group, per time point, *P<0.05.

Hsp70 expression has been documented to increase during the regeneration and re-growth period following muscle injury, suggesting its role in promoting muscle recovery [39]. In order to determine the requirement of Hsp70 for normal muscle regeneration and recovery following severe muscle injury, we injected TA muscles of *WT* and *Hsp70* ^−/−^ mice with 100 µl of 10 µM cardiotoxin. This standardized method of muscle injury is a highly reproducible model used in the study of muscle regeneration that induces widespread muscle fiber degeneration and necrosis in greater than 90% of muscle fibers [40], thereby limiting the variability in the extent of injury between animals that may occur using other models. Muscles were harvested and compared 1, 4, 16, 28 or 42 days following cardiotoxin injury. In order to first determine in our hands, the relative changes in Hsp70 gene expression in the days following injury we measured the mRNA levels of *Hspa1a* (which codes for Hsp70) 1, 4 and 16 days post-injury. As shown in Figure 2A, in *WT* mice Hspa1a mRNA was elevated ∼3.5-fold 1 day post-injury, unchanged 4 days post-injury and again elevated ∼2.5-fold 16 days post-injury, indicating a biphasic response in Hsp70 gene expression post-injury. Morphological analyses of injured muscles from *WT* and *Hsp70* ^−/−^ mice at these same time points post-injury via H&E staining of muscle-cross sections is shown in Figure 2B–D. As demonstrated in previous studies, 1 day following cardiotoxin injury muscles from *WT* mice showed significant mononuclear cell infiltration throughout the muscle and significant fiber degeneration and necrosis as evidenced by irregular eosinophilic staining and loss of muscle fiber integrity. In contrast, injured muscles from *Hsp70* ^−/−^ mice showed strikingly less infiltration by mononuclear cells compared to *WT*, even near vascular structures where infiltrating cells would be expected to be the most concentrated. Evidence for fiber degeneration was also visually less in *Hsp70* ^−/−^ mice compared to *WT* at this time point. Morphological analyses of muscles 4 days post-injury revealed that degenerating and/or necrotic muscle fibers were largely cleared in *WT* mice, and replaced by centrally nucleated myoblasts and other mononuclear cells, indicative of the early phase of muscle regeneration (Figure 2C). Although muscles from *Hsp70* ^−/−^ mice similarly showed centrally nucleated myoblasts and mononuclear cells at this time point, *Hsp70* ^−/−^ mice also displayed persisting necrotic fibers and abnormal basophilic staining throughout the muscle that was not evident in *WT*. Further analysis of muscles 16 days post-injury revealed restoration of normal muscle fiber architecture and regenerating fibers with centralized nuclei in *WT* mice (Figure 2D). In contrast, muscles from *Hsp70* ^−/−^ mice still presented with numerous necrotic muscle fibers and inflammatory lesions and showed visually smaller regenerating muscle fibers compared to *WT*. As shown in Figure 2E, the average CSA of regenerating muscle fibers in *Hsp70* ^−/−^ mice (945 µm^2^) was indeed significantly lower than that of WT mice (1524 µm^2^). Interestingly, the average number of regenerating myofibers was nearly 40% greater in *Hsp70* ^−/−^ mice compared to *WT* mice at this time point (Figure 2F). Presentation of this data as a fiber size frequency distribution (Figure 2E) demonstrates that the increased numbers of regenerating fibers in muscles from *Hsp70* ^−/−^ mice lie predominately in the CSA range of 250–750 µm^2^ (bin center  = 500). This significant increase in small regenerating fibers in muscles from *Hsp70* ^−/−^ mice found at this time point could be the result of ongoing cycles of muscle degeneration/regeneration. Alternatively, this finding could also indicate an impairment in myoblast fusion, since muscles from *Hsp70* ^−/−^ mice also had significantly lower numbers of fibers containing centralized nuclei in the larger CSA range (>1500 µm^2^).

### Hsp70^−/−^ mice do not show marked differences in myogenic markers post muscle-injury

**Figure 3:**
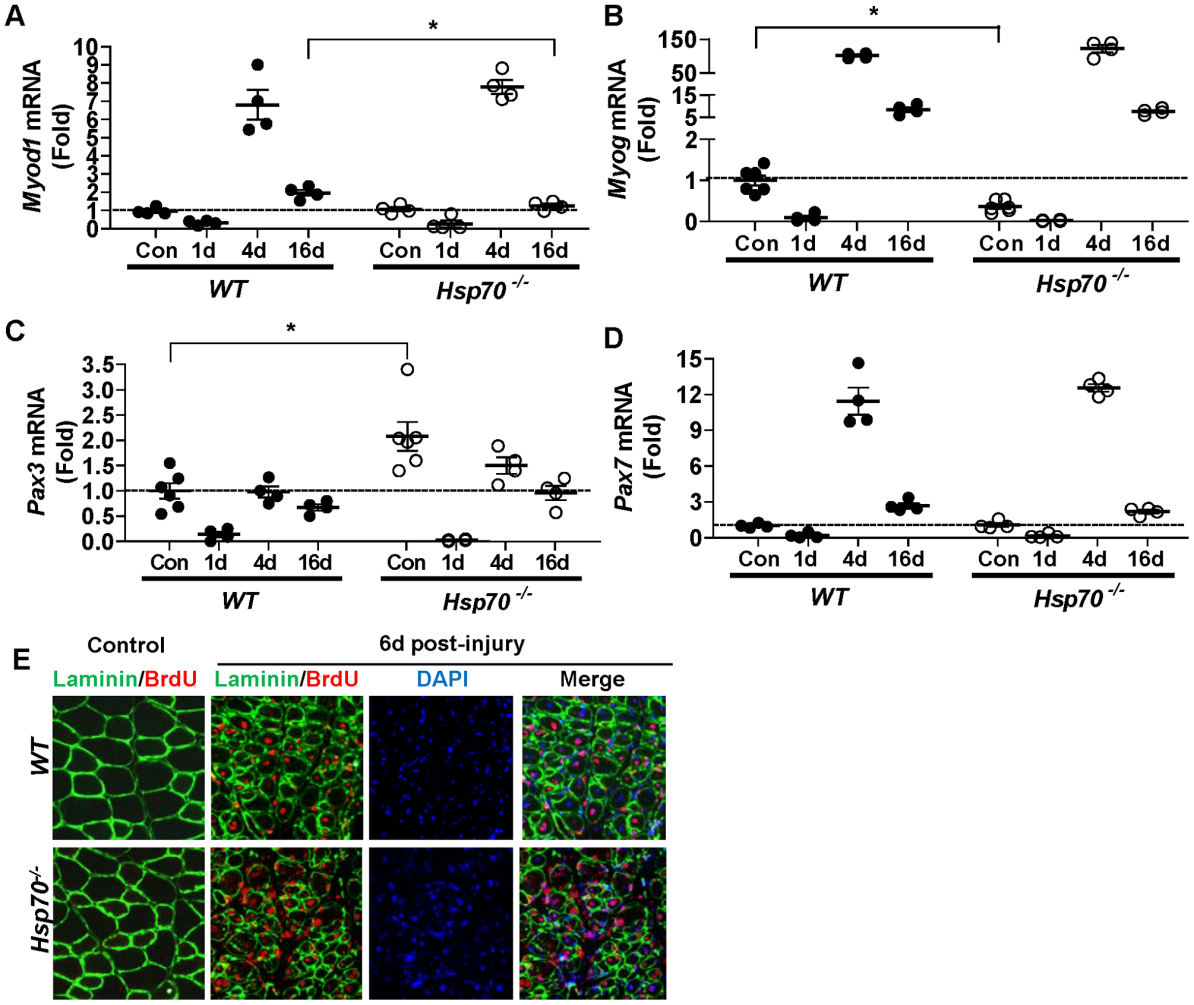
Injured muscles from *Hsp70* ^−/−^ mice are able to activate the myogenic program similar to *WT*. The gene expression of the myogenic transcription factors *Myod1* (A), *Myog* (B), *Pax3* (C) and *Pax7* (D) was measured in control muscles and injured muscles from *WT* and *Hsp70* ^−/−^ mice 1 day, 4 days and 16 days post cardiotoxin-injury. (E) BrdU incorporation into proliferating cells was assessed in control muscles and injured muscles of *WT* and *Hsp70* ^−/−^ mice during the first 6 days post- injury. Daily IP injections of BrdU were given starting 6 hours prior to muscle injury and muscles were harvested 6 days following injury. BrdU-positive nuclei were identified in muscle cross-sections using antibodies for BrdU (red) and laminin (green) to outline muscle fiber basement membranes. Injured muscles showing positive BrdU staining were co-stained with DAPI (blue) and the images merged to confirm the nuclear localization of BrdU (pink) inside of and surrounding myofibers. Numerical data represent mean ± SEM, and all data are representative of at least n = 4 mice/group, per time point, *P<0.05.

The impairments in muscle regeneration and recovery seen in muscles of *Hsp70* ^−/−^ mice may result from impairments in various aspects of the muscle degeneration/regeneration process. While muscles from *Hsp70* ^−/−^ mice were characterized by less mononuclear cell infiltration and muscle fiber degradation 1 day post-injury compared to *WT*, later time points indicated ongoing necrosis, sustained inflammation and deficits in muscle fiber regeneration. In order to better understand the mechanisms which may contribute to these regenerative deficits, we sought to first compare the gene expression of proteins which regulate satellite cell activation, proliferation and differentiation, and therefore muscle regeneration. We therefore measured the mRNA levels of myogenic differentiation 1 (*Myod1*), myogenin (*Myog*), paired box gene 3 (*Pax3*) and paired box gene 7 (*Pax7*) in uninjured (control) muscles of *WT* and *Hsp70* ^−/−^ mice and in injured muscles, 1 day, 4 days and 16 days following cardiotoxin injury. In control muscles we found that the levels of *Myod1* and *Pax7* were not statistically different between *WT* and *Hsp70* ^−/−^ mice. However, the levels of *Pax3* were significantly elevated in control muscles from *Hsp70* ^−/−^ mice compared to *WT*, and the levels of *Myog* significantly decreased in *Hsp70* ^−/−^ mice compared to *WT* (Figure 3A–D). These data indicate that under baseline conditions the myogenic program is indeed altered in muscles in the absence of Hsp70, which may explain in part, our earlier finding of altered myofiber CSA in *Hsp70* ^−/−^ mice.

Importantly, in response to cardiotoxin injury, the relative mRNA levels of *Myod1, Myog, Pax3* and *Pax7* were not statistically different between muscles from *WT* and *Hsp70* ^−/−^ mice at any time point post-injury, with the exception of *Myod1*. However, the levels of *Myod1* were only different in muscles of *WT* and *Hsp70* ^−/−^ mice at 16 days post-injury. At this time point the levels of MyoD1 remained elevated in injured muscles of WT mice by ∼100% over control muscles, whereas the levels of *Myod1* in injured muscles of *Hsp70* ^−/−^ mice were nearly back to baseline. Notably, the mRNA levels of these myogenic markers were highly comparable in muscles of *WT* and *Hsp70* ^−/−^ mice 4 days post-injury, which is a time point in which muscle satellite cell activation, proliferation and differentiation is at its peak [41]. These data therefore suggest that the ability of *Hsp70* ^−/−^ mice to activate the myogenic program is not significantly compromised.

**Figure 5:**
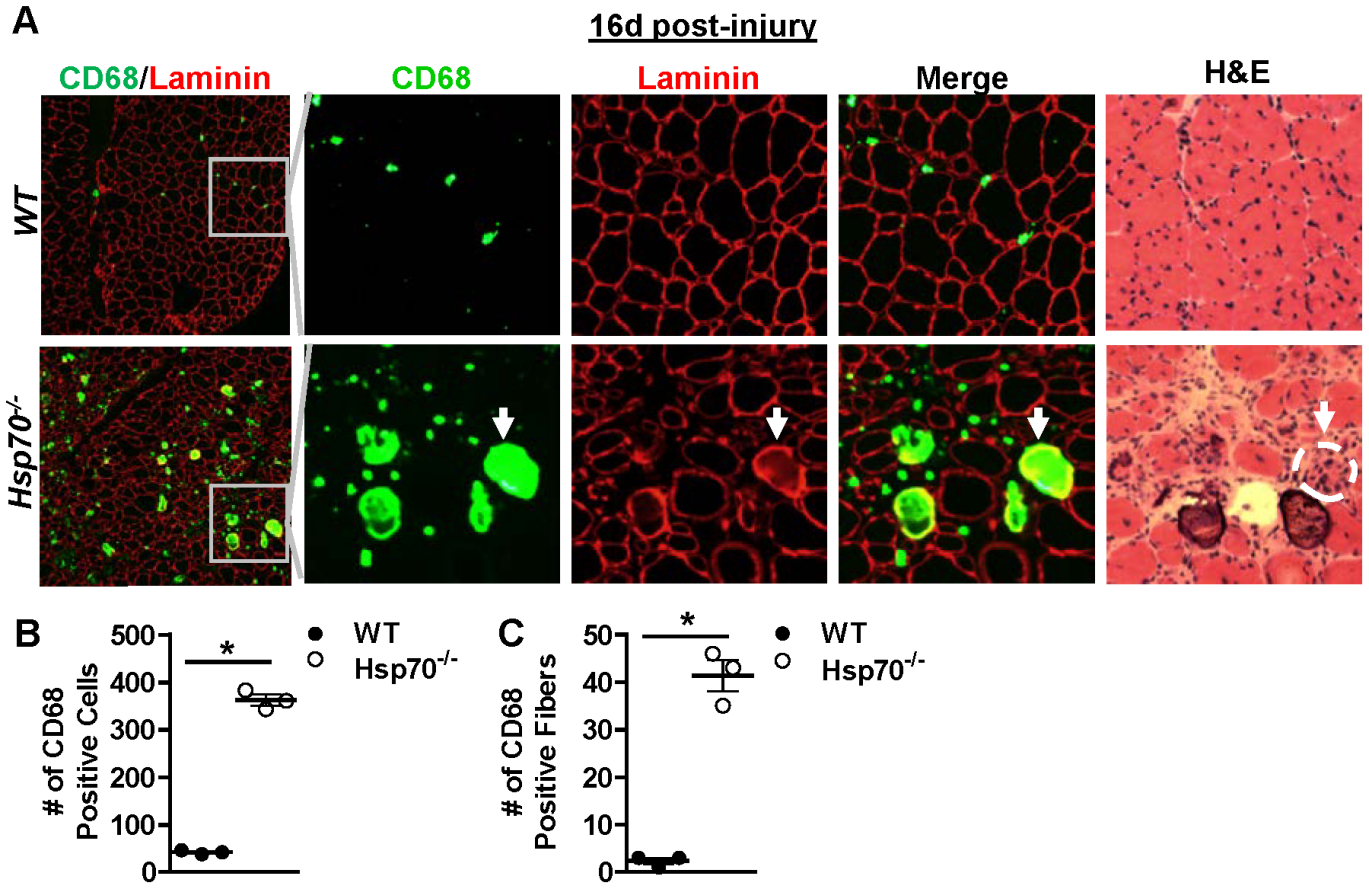
*Hsp70* ^−/−^ mice show sustained muscle inflammation 2 weeks post-injury. (A) Representative cross-sections from injured TA muscles from *WT* and *Hsp70* ^−/−^ mice 16 days post-injury stained with antibodies for CD68 to identify pro-inflammatory macrophages (green) and laminin to outline muscle fiber basement membranes (red). Magnification and separation of the merged images demonstrates the localization of CD68-positive macrophages to areas both surrounding and inside of muscle fibers. H&E staining of serial cross-sections further confirms that muscle fibers heavily infiltrated by mononuclear cells on H&E-stain are also positive for CD68-positive macrophages (white arrows denote a corresponding fiber in serial sections, which is also outlined in white in the H&E-stained image). (B) Quantification of the number of CD68-positive macrophages located outside of the basal lamina. (C) Quantification of the number of muscle fibers showing CD68-positive staining (macrophage infiltration) underneath the basal lamina. Numerical data represent the mean ± SEM, and all data are representative of at least n = 3 mice/group, *P<0.05.

In order to take a different approach to measure myogenesis, we performed BrdU assays, *in vivo*, to determine the ability of myogenic progenitor cells in skeletal muscles of *WT* and *Hsp70* ^−/−^ mice to proliferate following injury. *WT* and *Hsp70* ^−/−^ mice were injected IP with BrdU (which is incorporated into the DNA of proliferating cells) once daily beginning 6 hrs prior to cardiotoxin-injury. Six days post-injury, the injured TA and the contralateral, uninjured (control) TA were harvested for histological analyses. Muscle cross-sections from control and injured TA muscles were incubated with antibodies for BrdU and laminin, to identify BrdU-positive nuclei underneath the basal lamina of muscle fibers (Figure 5E). Cross-sections from injured TA muscles showing positive BrdU staining were also counterstained with DAPI, to confirm the nuclear location of BrdU. Control muscles from both *WT* and *Hsp70* ^−/−^ mice showed few BrdU-positive nuclei. In contrast, injured muscles from *WT* and *Hsp70* ^−/−^ mice showed abundant levels of BrdU nuclear staining underneath the basal lamina of muscle fibers, which is indicative of muscle satellite cell proliferation. Some BrdU-positive nuclei in muscle cross-sections are likely those of inflammatory cells, fibrotic cells and other proliferating cells present in regenerating skeletal muscle. However, the abundant localization of BrdU-positive nuclei underneath the basal lamina and in the center of myofibers in both *WT* and *Hsp70* ^−/−^ mice strongly suggests that both strains of mice are capable of successful muscle satellite cell proliferation and differentiation. Therefore, our findings using BrdU-labeling of proliferating cells support our gene expression data, which suggest that *Hsp70* ^−/−^ mice do not have significant deficits in their ability to activate the myogenic program, despite the deficits in regenerating fiber size seen in these mice at later time points.

### Muscles from Hsp70^−/−^ mice show decreased markers of immune cell recruitment 24 hours post-injury

**Figure 4:**
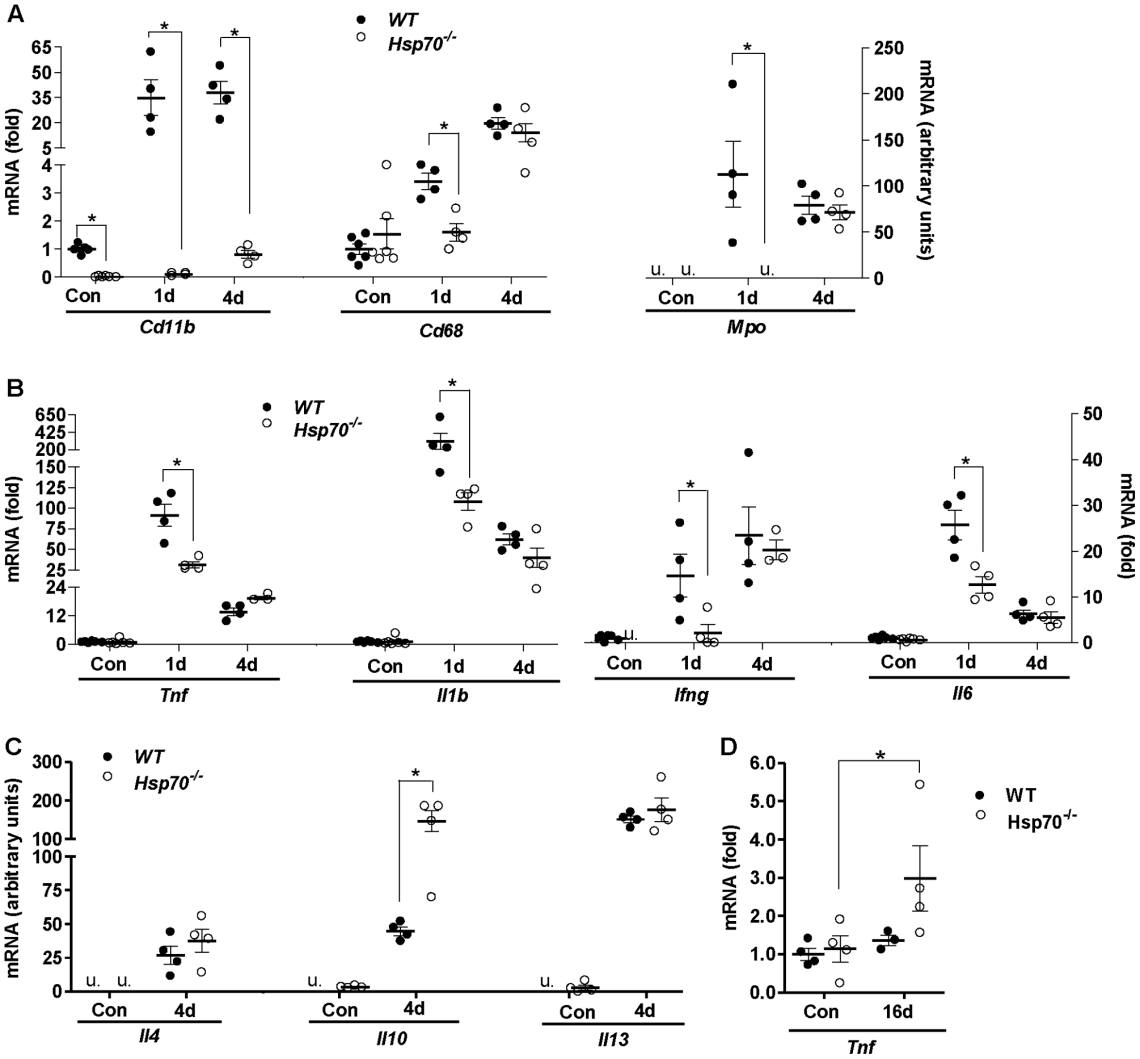
*Hsp70* ^−/−^ mice have a significantly delayed immune response to injury. The relative mRNA levels of immune cell markers *Cd11b*, *Cd68* and *Mpo* (A) and pro-inflammatory cytokines *Tnf*, *Ifng, Il1b* and *Il6* (B) were measured in control muscles and injured muscles from *WT* and *Hsp70* ^−/−^ mice 1 day and 4 days post cardiotoxin-injury. (C) The relative mRNA levels of anti-inflammatory cytokines *IL4*, *IL10* and *IL13* in control and injured muscles from *WT* and *Hsp70* ^−/−^ mice 4 days post cardiotoxin-injury. (D) The relative mRNA levels of *Tnf* in muscles from *WT* and *Hsp70* ^−/−^ mice 16 days post-injury. All data represent mean ± SEM, and n =  at least 4 mice per group, per time point, *P<0.05, u =  undetected.

Successful activation of the myogenic program following muscle injury is not the only determinant of efficient muscle regeneration. Activation and subsequent resolution of the immune response is also a critical determinant of successful muscle remodeling following injury [39], [42]. Since compared to *WT*, *Hsp70* ^−/−^ mice showed significantly less mononuclear cell infiltration in H&E-stained muscle cross-sections 1 day post-injury, and persisting necrotic muscle fibers 4 days post-injury, we hypothesized that muscles from *Hsp70* ^−/−^ mice would show an altered gene expression profile of inflammatory cell markers and cytokines involved in the inflammatory response to injury. We therefore measured the mRNA levels of genes expressed specifically by immune cell populations involved in the innate immune response to injury in muscles of *WT* and *Hsp70* ^−/−^ mice 1 day and 4 days post muscle injury. We chose to measure *Cd11b* (expressed by leukocytes involved in the innate immune response including neutrophils, monocytes and macrophages, and is also known as integrin alpha M, macrophage-1 antigen or complement receptor 3), *Cd68* (expressed by pro-inflammatory macrophages) and myeloperoxidase (*Mpo)* (expressed by neutrophils – which are the first immune cells to infiltrate damaged tissue following injury). As expected, 1 day post-injury muscles from *WT* mice showed evidence for immune cell infiltration as measured by increases in the mRNA levels of *Cd11b*, *Cd68*, and *Mpo* compared to control muscles (Figure 4A). In contrast both *Cd11b* and *Mpo* were virtually undetectable in control muscles of *Hsp70* ^−/−^ mice, and were not increased in injured muscles 1 day following injury. Further the mRNA levels of *Cd68* were also unchanged in injured muscles of *Hsp70* ^−/−^ mice 1 day post-injury. These data support our histological findings at this time point, which indicate a significant deficit in immune cell infiltration into injured muscles of *Hsp70* ^−/−^ mice during the first 24 hours following injury. Analyses of these markers in muscles 4 days post-injury showed a similar deficit in the mRNA levels of *Cd11b* in *Hsp70* ^−/−^ mice compared to *WT*. However, the mRNA levels of *Cd68* and *Mpo* were no longer significantly different between injured muscles of *WT* and *Hsp70* ^−/−^ mice at this time point. These data suggest that in damaged muscles of *Hsp70* ^−/−^ mice, the early infiltration of immune cell populations expressing *Cd68* (pro-inflammatory macrophages) and *Mpo* (neutrophils) is significantly delayed, reaching levels comparable to *WT* by the fourth day following injury. However, since *Cd11b* is expressed by both neutrophils and macrophages, yet this transcript remained absent in muscles of *Hsp70* ^−/−^ mice, this may indicate that the intrinsic ability of immune cells to increase *Cd11b* transcription may be compromised in *Hsp70* ^−/−^ mice.

Since immune cell infiltration into muscle propagates the inflammatory response to injury, in part, through their increased expression and secretion of pro-inflammatory cytokines, we further measured the mRNA levels of *Tnf*, *Il1b, Ifng* and *Il6* in control and injured muscles 1 day and 4 days post-injury. As expected, muscles from *WT* mice showed significant elevation of these transcripts 1 day post-injury. Compared to WT, muscles from *Hsp70* ^−/−^ mice showed significantly attenuated increases in the mRNA levels of these pro-inflammatory cytokines 1 day following injury (Figure 4B), which is in line with our finding of reduced markers of immune cell infiltration in these mice at this time point. Measurement of these transcripts in muscles 4 days post-injury demonstrated comparable levels of *Il1b, Ifng* and *Il6* mRNA between *WT* and *Hsp70* ^−/−^ mice, and increased levels of *Tnf* mRNA in *Hsp70* ^−/−^ mice compared to *WT*. Pro-inflammatory macrophages are major sources of these pro-inflammatory cytokines, and are the predominant immune cell population present in injured muscle 4 days following injury [43]. Therefore, our finding that the mRNA levels of the pro-inflammatory macrophage-specific marker, *Cd68*, and pro-inflammatory cytokines are comparable in muscles of *WT* and *Hsp70* ^−/−^ mice 4 days post-injury are in agreement, and indicate that the level of muscle inflammation may be similar between *WT* and *Hsp70* ^−/−^ mice by the fourth day post-injury. Again this is in stark contrast to our findings in muscles 1 day post-injury, when significant deficits in immune cell markers and pro-inflammatory cytokines were evident in muscles from *Hsp70* ^−/−^ mice.

We next measured the gene expression of the anti-inflammatory cytokines Il4, Il10 and Il13 in muscles 4 days post-injury, which play important roles in shifting the muscle microenvironment from a pro-inflammatory to pro-healing environment during the regenerative phase following injury [43]. As shown in Figure 4C, the mRNA levels of *Il4*, *Il10* and *Il13* were each significantly elevated in injured muscles of *WT* and *Hsp70* ^−/−^ mice 4 days post-injury. However, the levels of *Il10* were significantly greater in *Hsp70* ^−/−^ mice compared to *WT* at this time point. Since both *Tnf* and *Il10* were significantly elevated above *WT* in muscles from *Hsp70* ^−/−^ mice 4 days post-injury, we further determined whether these cytokines remained increased above *WT,* 16 days post-injury. Even at low dilutions of cDNA, we were unable to consistently detect *Il10* expression in muscles from *WT* and *Hsp70* ^−/−^ mice 16 days post-injury. In contrast, the mRNA levels of *Tnf* remained significantly elevated in muscles of both *WT* and *Hsp70* ^−/−^ mice 16 days post-injury, with increased expression of Tnf in *Hsp70* ^−/−^ mice compared to *WT*. Together these gene expression data on immune cell markers and inflammatory cytokines indicate that muscles from *Hsp70* ^−/−^ mice have a significantly delayed skeletal muscle inflammatory response in the first 24 hours following muscle injury which may contribute to increased inflammation and necrosis and impaired regeneration seen in these mice at later time points post-injury.

### Pro-inflammatory macrophages persist in Hsp70^−/−^ mice 2 weeks post-injury

Since significant muscle fiber necrosis and increased expression of the pro-inflammatory cytokine, Tnf, were evident in injured muscles of *Hsp70* ^−/−^ mice 16 days post-injury, we hypothesized that pro-inflammatory macrophages would also be abnormally elevated in muscles at this time point. Cross-sections taken from injured muscles from *WT* and *Hsp70* ^−/−^ mice 16 days post-injury were therefore co-incubated with antibodies for CD68 to label pro-inflammatory macrophages, and antibodies for laminin to label muscle fiber basement membranes. As shown in Figure 5A, significant CD68-positive staining was seen across entire cross-sections of muscles *Hsp70* ^−/−^ mice, but not *WT* mice. Magnification and separation of the overlayed CD68- and laminin-stained images in Figure 5B further demonstrates that CD68-positive macrophages are present both surrounding, and inside of muscle fibers in muscles from *Hsp70* ^−/−^ mice. Comparison of these CD68-stained areas of the muscle to serial sections stained with H&E demonstrates that muscle fibers which appear infiltrated by numerous mononuclear cells on H&E stain also stain diffusely for CD68, demonstrating that these necrotic fibers are indeed strongly infiltrated by pro-inflammatory macrophages. Furthermore, the abnormal basophilic-stained fibers seen on H&E-stain in muscles from *Hsp70* ^−/−^ mice also stain positively for CD68, which indicates that this abnormal staining is associated with increased muscle fiber inflammation. The average number of CD68-positive puncti (counted as macrophages) in muscle cross-sections located outside the basal lamina were calculated separately from the average number of muscle fibers showing CD68-positive macrophages underneath the basal lamina. Compared to WT, injured muscles from *Hsp70* ^−/−^ mice showed an approximate 7-fold increase in the number of CD68-positive macrophages surrounding muscle fibers (Figure 5C), and a 10-fold increase in the number of muscle fibers infiltrated by CD68-positive macrophages (Figure 5D). Collectively, our gene expression and histological data indicate that Hsp70 is necessary for both timely activation of the inflammatory response to muscle injury, as well as timely resolution of muscle inflammation that is expected to occur during the regenerative phase.

### Calcification in muscles from Hsp70^−/−^ mice following injury

**Figure 6:**
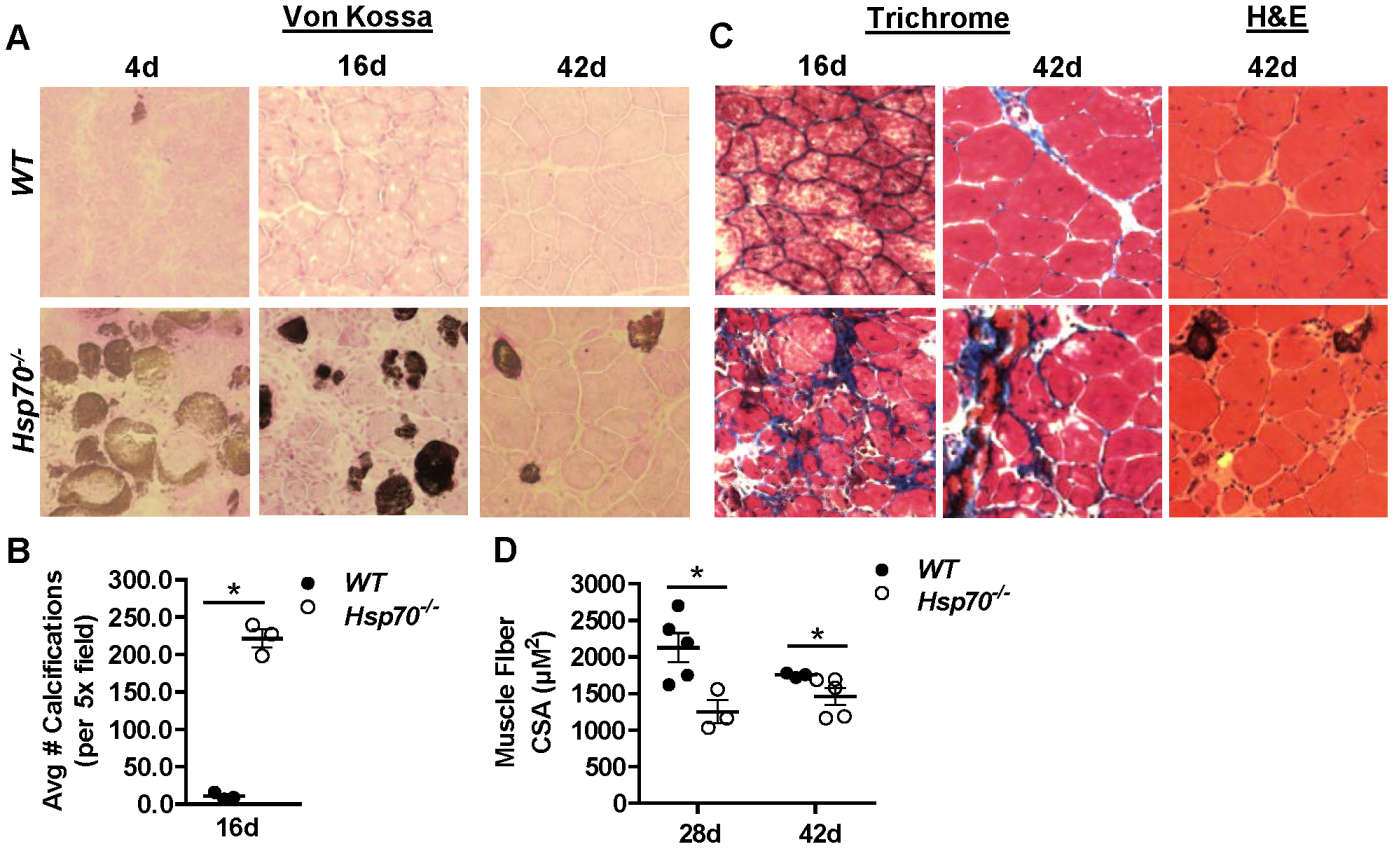
Calcifications, collagen deposition and impaired regeneration in *Hsp70* ^−/−^ mice up to 6 weeks post-injury. (A) Representative cross-sections from injured muscles of *WT* and *Hsp70* ^−/−^ mice 4 days, 16 days and 42 days post cardiotoxin-injury stained using Von Kossa procedure which stains calcifications brown. (B) The average number of calcifications across entire muscle cross-sections was calculated in muscles 16 days post-injury. (C) Cross-sections of injured muscles from *WT* and *Hsp70* ^−/−^ mice 16 days and 42 days post-injury were stained using Masson's Trichrome procedure, which stains muscle cytoplasm red and collagen blue. (D) The average fiber CSA was calculated in injured muscles from *WT* and *Hsp70* ^−/−^ mice 28 days and 42 days post cardiotoxin-injury. Numerical data represent mean ± SEM, and all data are representative of at least n = 3 mice/group per time point, *P<0.05.

Since muscles from *Hsp70* ^−/−^ mice showed persistent fiber degeneration/necrosis and increased pro-inflammatory markers at both 4 and 16 days post-injury, we hypothesized that this may be related to increased levels of calcium, which is a known regulator of pathways involved in inflammation and fiber degeneration [36]. To test this we stained muscle cross-sections using Von Kossa procedure, which stains calcium deposits dark brown. As shown in Figure 6A, *WT* muscles do not show significant calcium deposition. In contrast, muscles from *Hsp70* ^−/−^ mice showed pronounced calcium deposition as early as 4 days post-injury which remained in muscle cross-sections up to 42 days post-injury. Quantification of these calcifications in entire muscle cross-sections 16-days post-injury showed on average, greater than 200 calcifications present in muscles from *Hsp70* ^−/−^ mice, compared to less than 10 in muscles from *WT* mice (Figure 6B).

### Muscles from Hsp70^−/−^ mice show impaired muscle recovery up to 6-weeks post-injury

Persistent levels of inflammation and failed regeneration in skeletal muscle following injury is known to result in replacement of muscle tissue with fibrotic tissue. Based on our findings that muscles from *Hsp70* ^−/−^ mice recovering from cardiotoxin-injury show sustained presence of pro-inflammatory macrophages, calcifications and smaller regenerating fibers, we hypothesized that these muscles would also show increased collagen deposition. Trichrome staining of muscle cross-sections 16 days and 42 days post cardiotoxin-injury demonstrates visual increases in collagen deposition surrounding muscle fibers in muscles from *Hsp70* ^−/−^ mice when compared to *WT* at both time points (Figure 6C).

Since a significant deficit in regenerating fiber CSA was present in muscles from *Hsp70* ^−/−^ mice when compared to muscles of *WT* mice 16 days post-injury, we further quantified total fiber CSA in *WT* and *Hsp70* ^−/−^ mice at both 28 and 42 days post-injury to determine if fiber CSA in *Hsp70* ^−/−^ mice would eventually reach that of *WT*. As shown in Figure 6D, fiber CSA in *Hsp70* ^−/−^ mice remained significantly below *WT* at both 28 days (*WT*  = 2130 µm^2^ and *Hsp70* ^−/−^  = 1251 µm^2^) and 42 days (*WT*  = 1754 µm^2^ and *Hsp70* ^−/−^  = 1461 µm^2^) post-injury. Collectively these data demonstrate significant impairments in muscle regeneration and recovery in *Hsp70* ^−/−^ mice that persist up to 6 weeks post-injury.

### Restoration of Hsp70 in skeletal muscle prior to injury rescues regenerative deficits in Hsp70^−/−^ mice

**Figure 7:**
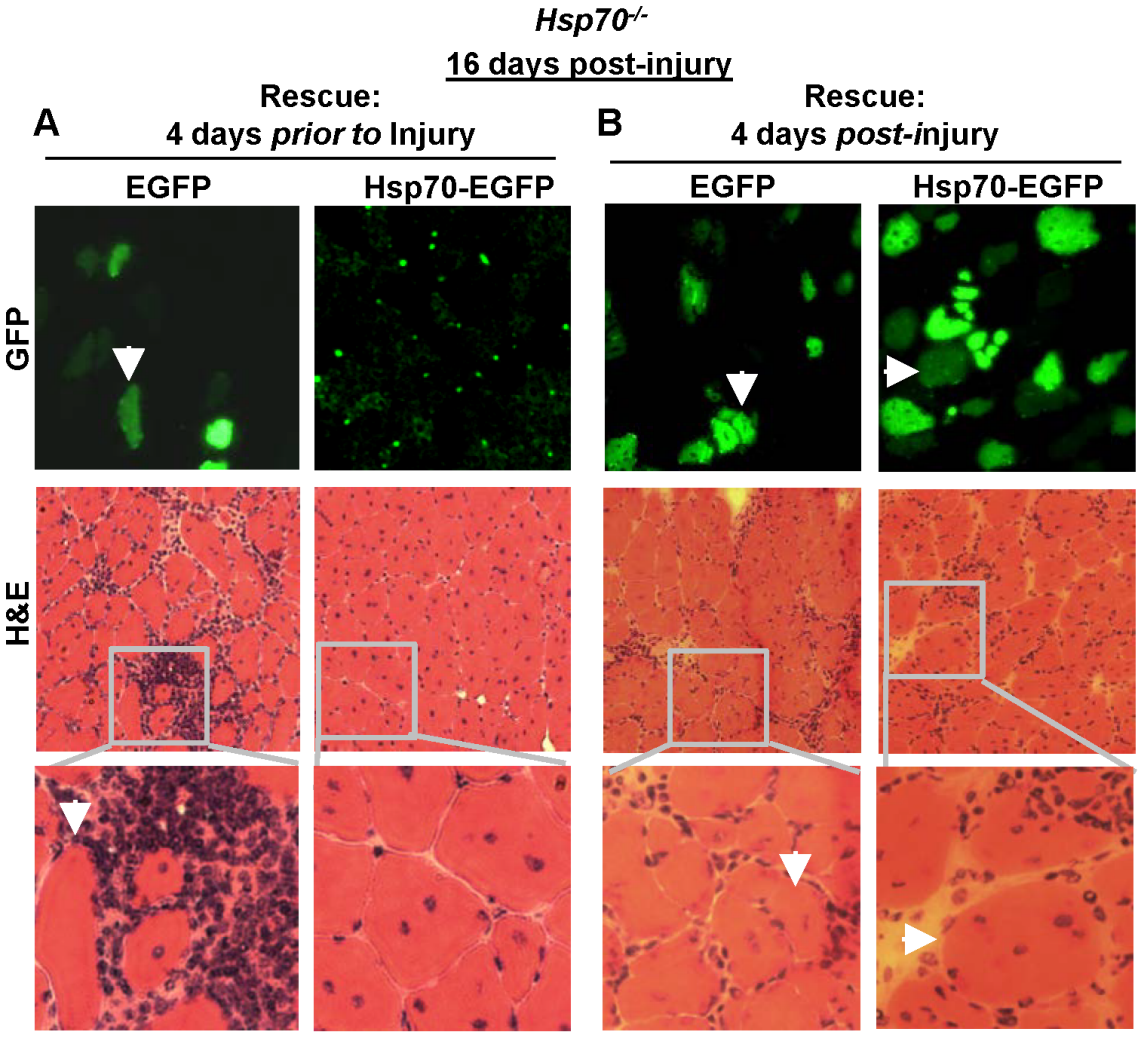
Restoration of Hsp70 in skeletal muscle rescues regenerative deficits in *Hsp70* ^−/−^ mice. TA muscles of *Hsp70* ^−/−^ mice were electroporated with either EGFP or Hsp70-EGFP expression plasmids 4 days prior to cardiotoxin-injury (A) or 4 days following cardiotoxin injury (B). Muscles were harvested 16 days post cardiotoxin-injury and serial cross-sections viewed under a GFP filter to visualize transfected fibers and H&E-stained to determine muscle morphology. Representative images are shown for each group and are representative of n = 4 mice per group. Arrows indicate corresponding fibers in serial cross-sections.

Since Hsp70 whole-body knockout mice were used in the current study, the deficits in muscle regeneration and recovery may be related to the lack of Hsp70 in any cell type involved in muscle regeneration. To determine whether reintroduction of Hsp70 specifically in skeletal muscle fibers could restore the deficits in regeneration in *Hsp70* ^−/−^ mice, we performed rescue experiments reintroducing Hsp70 tagged to EGFP, or EGFP as the control, into muscles of *Hsp70* ^−/−^ mice using expression plasmid injection and electroporation. This method has been used extensively by numerous lab groups to induce transgene expression specifically in skeletal muscle fibers [8], [14], [44], [45]. Hsp70-EGFP or EGFP was electroporated into TA muscles of *Hsp70* ^−/−^ mice either 4 days prior to cardiotoxin-injury or 4 days post cardiotoxin-injury. We chose these time points to compare the relative importance of Hsp70 expressed in skeletal muscle fibers at the onset of muscle injury during the inflammatory phase versus the importance of Hsp70 during the regenerative phase of muscle recovery. Since we observed a blunted immune response in muscles of *Hsp70* ^−/−^ mice 1 day post-injury, we hypothesized that reintroduction of Hsp70 into the muscle prior to muscle injury would provide greater rescue than experiments reintroducing Hsp70 4 days post-injury. As shown in Figure 7A, electroporation of the control plasmid, EGFP, into muscles of *Hsp70* ^−/−^ mice prior to cardiotoxin-injury did not rescue the deficits in muscle regeneration/recovery in these mice, as indicated by significant presence of inflammatory cells and small regenerating fibers when visualized 16 days post-injury. In contrast, muscles of *Hsp70* ^−/−^ mice electroporated with Hsp70-EGFP prior to cardiotoxin-injury were almost completely devoid of inflammatory lesions 16 days post-injury, and contained visually larger regenerating fibers than muscles *of Hsp70* ^−/−^ mice electroporated with EGFP, and showed several centralized myonuclei per myofiber. Interestingly, visualization of EGFP versus Hsp70-EGFP fluorescence in electroporated muscles revealed significant presence of EGFP, but not Hsp70-EGFP in muscle fibers 16 days post-injury. EGFP and Hsp70-EGFP normally show similar transfection efficiency and levels of fluorescence in skeletal muscle following electroporation ([8] and Figure 7B). Therefore, the reduced fluorescence/expression of Hsp70-EGFP (but not EGFP) following muscle injury suggests that Hsp70 may be preferentially released from muscle fibers following injury. Alternatively, the introduction of Hsp70-EGFP (but not EGFP) into muscles of *Hsp70* ^−/−^ mice may restore the ability of muscle to remove damaged and necrotic cellular debris, thus concurrently resulting in enhanced removal of Hsp70-EGFP in the damaged tissue.

In separate experiments we electroporated muscles from *Hsp70* ^−/−^ mice with Hsp70-EGFP, or EGFP, 4 days following cardiotoxin-injury to determine the importance of Hsp70 during the regenerative phase of muscle recovery. As shown in Figure 7B, muscle fibers positive for Hsp70-EGFP were visually larger than EGFP-positive muscle fibers, and showed several centralized nuclei per transfected myofiber. These data provide clear evidence that Hsp70 expressed in regenerating muscle fibers is necessary for fiber regrowth, and further suggest that Hsp70 may regulate myoblast fusion. Furthermore, although all *Hsp70* ^−/−^ mice electroporated with Hsp70-EGFP (i.e. transfected prior to and following injury) showed significant rescue of regenerating muscle fiber size, the persisting necrotic fibers, inflammatory lesions and indications for fibrosis in injured muscles of *Hsp70* ^−/−^ mice were only prevented when Hsp70-EGFP was introduced into the muscle *prior to* injury. This data suggests that Hsp70 plays an especially critical role in the first four days following severe injury to subsequently promote efficient muscle regeneration and recovery. Since the timely activation of inflammatory processes following severe muscle injury is necessary for efficient muscle regeneration, our findings thus far collectively indicate that Hsp70 promotes efficient muscle regeneration and recovery, in part, through activating the early inflammatory response to muscle injury.

### Simulation of Hsp70 extracellular release at the onset of muscle injury restores the early inflammatory response in muscles of Hsp70^−/−^ mice

**Figure 8:**
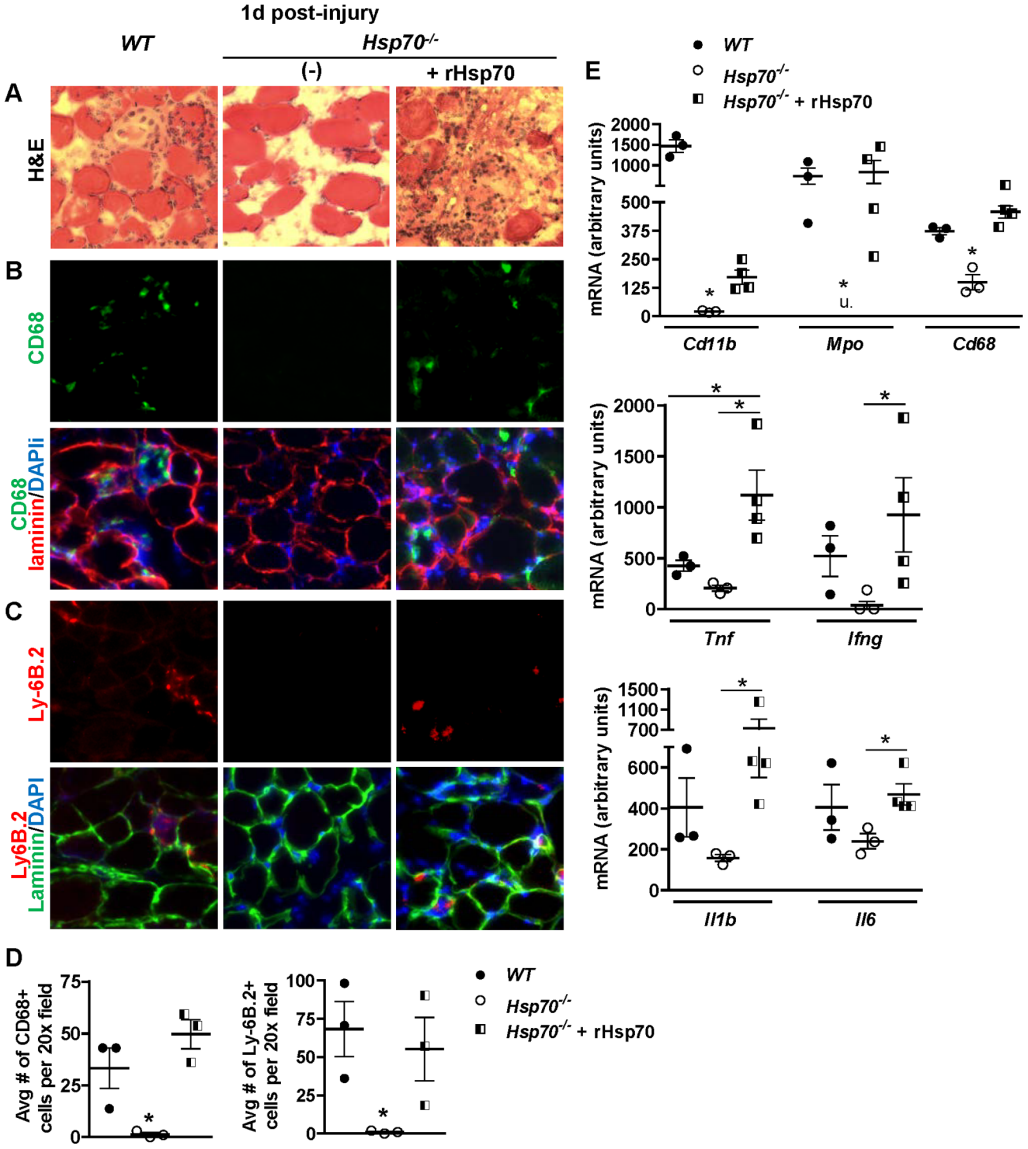
Simulation of Hsp70 extracellular release restores deficits in inflammatory response to muscle injury in *Hsp70* ^−/−^ mice. TA muscles of *WT* and *Hsp70* ^−/−^ mice were injected with cardiotoxin only or cardiotoxin + rhHsp70 and harvested 1 day post-injury for histological and gene expression analyses of pro-inflammatory markers. (A) Representative muscle cross-sections stained with H&E. (B) Representative muscle cross-sections incubated with antibodies for CD68 to label pro-inflammatory macrophages (green), and laminin to outline muscle fiber basement membranes (red) and co-stained with DAPI to identify cell nuclei (blue). (C) Representative muscle cross-sections incubated with antibodies for Ly-6B.2 to label polymorphonuclear cells (e.g. neutrophils) (red) and laminin to outline muscle fiber basement membranes (green), and co-stained with DAPI to identify cell nuclei (blue). (D) The average number of CD68+ and Ly-6B.2+ cells per 20x field in skeletal muscle cross-sections 1 day post-injury. (E) The mRNA levels of immune cell markers *Cd11b*, *Mpo* and *Cd68*, and pro-inflammatory cytokines *Tnf, Ifng*, *Il1b* and *Il6* in muscles 1 day post-injury. Numerical data represent mean ± SEM, and all data are representative of at least n = 3 mice/group. *Significantly different from all other groups unless otherwise noted, P<0.05, u =  undetected.

Studies have previously demonstrated that Hsp70 can be released into the extracellular microenvironment following cellular injury to act as a danger signal. Indeed, the extracellular form of Hsp70 has been shown in numerous studies to participate in both the innate and adaptive immune responses through binding and activating receptor-mediated signal transduction pathways in immune cells [46]. Importantly, the release of muscle fiber-derived proteins into the extracellular environment following muscle injury is believed to play an important role in activating and recruiting inflammatory cells to the site of muscle injury [42]. Based on the blunted immune response in muscles of *Hsp70* ^−/−^ mice following muscle injury, we therefore hypothesized that local release of Hsp70 into the extracellular microenvironment following muscle injury plays a key role in activating the innate immune response to injury. To test this theory, we injected recombinant Hsp70 (rHsp70) into TA muscles of *Hsp70* ^−/−^ mice at the time of muscle cardiotoxin-injury to simulate Hsp70 extracellular release. One day later, injured muscles were harvested and compared to cardiotoxin-injured muscles not receiving rHsp70. As shown in Figure 8A, H&E-staining of muscle cross-sections demonstrates that the decreased inflammatory cell infiltration observed in *Hsp70* ^−/−^ mice compared to *WT* was restored when rHsp70 was injected into the muscle at the time of muscle injury. Further immunostaining for pro-inflammatory macrophages using an antibody for CD68 (Figure 8B), and for polymorphonuclear cells (i.e. neutrophils) using an antibody for Ly-6B.2 (Figure 8C), confirmed the presence of both immune cell populations in injured muscles from *WT* mice, and their absence in injured muscles from *Hsp70* ^−/−^ mice. Treatment with rHsp70 restored both immune cell populations to injured muscles of *Hsp70* ^−/−^ mice to similar levels found in *WT*, which is quantified in Figure 8D. To substantiate these histological data showing rescue of the skeletal muscle immune response in *Hsp70* ^−/−^ mice treated with rHsp70, we further measured and compared the gene expression of immune cell markers and pro-inflammatory cytokines in muscles from these same groups 1 day post-injury. As shown in Figure 8E, the reduced and/or undetected mRNA levels of *Cd11b*, *Cd68, Mpo*, *Tnf*, *Ifng*, *Il1b* and *Il6* in injured muscles from *Hsp70* ^−/−^ mice compared to *WT* were all increased via injection of rHsp70. With the exception of *Cd11b*, the mRNA levels of these pro-inflammatory markers were each restored to values comparable to *WT*. Further, the mRNA levels of *Tnf* were significantly greater in *Hsp70* ^−/−^ muscles treated with rHsp70 when compared to *WT*. Collectively these data indicate that the extracellular form of Hsp70 regulates the early inflammatory response to skeletal muscle injury, and supports the notion that the active secretion and/or passive release of Hsp70 from damaged cells in muscle tissue plays a key role in the timely activation and recruitment of immune cells to the injury site.

### Decreased muscle damage following muscle reloading injury in Hsp70^−/−^ mice

**Figure 9:**
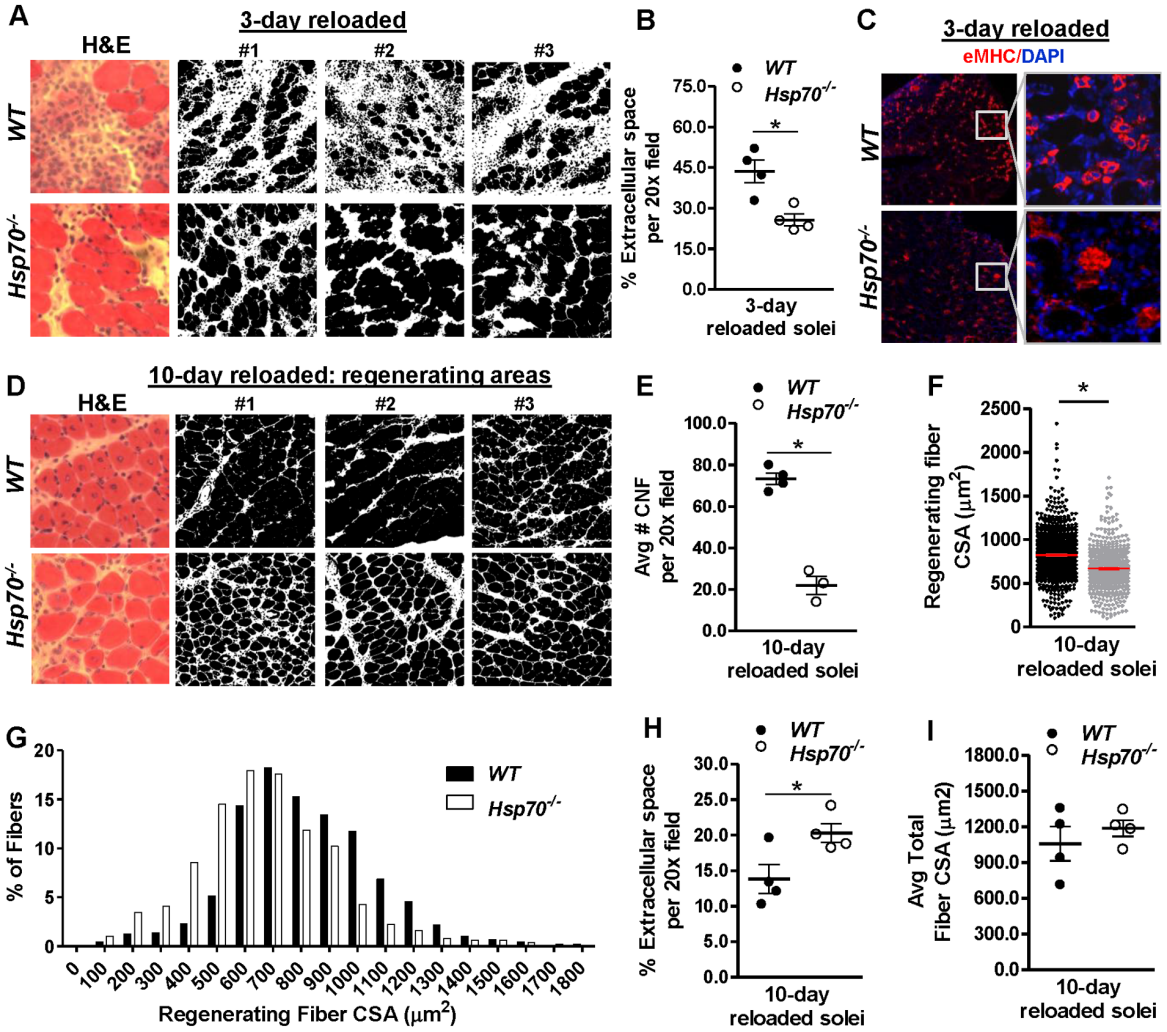
Decreased muscle damage but impaired fiber regeneration in *Hsp70* ^−/−^ mice following muscle reloading-injury. Muscle reloading-injury was induced in soleus muscles of *WT* and *Hsp70* ^−/−^ mice via bilateral hind limb cast immobilization for 10 days followed by cast-removal and reambulation for 3 or 10 days. (A & D) Representative bright field and binary-processed images taken from H&E-stained skeletal muscle cross-sections from 3-day reloaded muscles (A) and 10-day reloaded muscles (D). Images from 3 different mice per group are shown. (B and H) The percentage of extracellular tissue in H&E-stained cross-sections of 3-day reloaded muscles (B) and 10-day reloaded muscles (H) was approximated via calculating the percentage of white area per 20x field in binary-converted images. (C) Representative cross-sections from 3-day reloaded muscles stained with embryonic Myosin Heavy Chain (eMHC) and co-stained with DAPI to identify regenerating areas of the muscle. (E–G) The average number (E) and CSA (F) of regenerating fibers containing centralized nuclei (CNF) were calculated in 10-day reloaded muscles and is also shown in a fiber size frequency distribution (G). (I) The average fiber CSA of all fibers (regenerating and non-regenerating fibers) was calculated in 10-day reloaded muscles. Numerical data represent mean ± SEM, and all data are representative of at least n = 4 mice/group per time point, *P<0.05.

Data collected thus far using the standardized model of cardiotoxin-induced muscle injury demonstrates that Hsp70 is necessary for the normal immune response to skeletal muscle injury, and for efficient regeneration and recovery. To further test these findings in a more physiologically relevant model of mechanical muscle injury, we utilized a model of muscle reloading injury, also referred to as modified muscle use [47]. In our model, muscle disuse was induced in *WT* and *Hsp70* ^−/−^ mice through bilateral hind limb cast-immobilization for 10 days with the ankle in the plantar flexed position, followed by cast removal and reambulation for either 3 or 10 days. This protocol induces mechanical damage in the soleus muscle during the reambulation/reloading period, as demonstrated previously [48]. Soleus muscles were therefore removed following the 3 day or 10 day reloading period and processed for morphological analyses. As shown in Figure 9A, following 3 days of reloading, H&E-stained muscle cross sections from *WT* mice showed significant muscle reloading damage, as indicated by inflammatory cell infiltration, loss of muscle fibers, and early stage muscle regeneration. In contrast, muscles from *Hsp70* ^−/−^ mice showed significantly less myofiber damage, which was associated with decreased presence of mononuclear cell infiltration compared to *WT*. Quantification of the muscle fiber area versus extracellular tissue in muscles at this time point confirmed that *Hsp70* ^−/−^ mice were significantly protected from the loss of muscle fiber tissue in response to reloading (Figure 9B). To further compare the relative level of regeneration in 3-day reloaded soleus muscles from *WT* and *Hsp70* ^−/−^ mice, we stained muscle cross-sections for embryonic myosin heavy chain (eMHC), which is expressed by regenerating myoblasts and from the nuclei of regenerating myofibers derived from satellite cell activation, differentiation, and fusion. Cross-sections were also co-stained with DAPI, to identify cell nuclei. As shown in Figure 9C, both *WT* and *Hsp70* ^−/−^ mice showed significant eMHC-staining. However, magnification of these images demonstrates differential localization of eMHC-staining in 3-day reloaded solei from *WT* and *Hsp70* ^−/−^ mice. *WT* mice showed abundant staining of eMHC to mononuclear cells (myoblasts), while *Hsp70* ^−/−^ mice showed predominant localization of eMHC staining inside of muscle fibers, and relatively few eMHC-positive myoblasts. This staining pattern of eMHC fits with our finding that reloaded muscles from *Hsp70* ^−/−^ mice show less muscle fiber damage/degradation compared to *WT*, since decreased muscle damage would presumably result in a reduced need for satellite cell activation and proliferation to replace damaged areas of the muscle.

We subsequently analyzed the morphology of muscles following 10 days of reloading via H&E staining of muscle cross-sections (Figure 9D). We found that *WT* mice had largely regained normal muscle fiber architecture, though most myofibers were still undergoing fiber regeneration as indicated by the central location of myofiber nuclei. Muscles from *Hsp70* ^−/−^ mice also showed relatively normal muscle fiber architecture, and had substantially fewer regenerating myofibers containing centralized nuclei compared to *WT* (Figure 9E). This finding fits with the data collected in 3-day reloaded muscles, which showed evidence for decreased muscle reloading damage and activation of regenerative processes in muscles from *Hsp70* ^−/−^ mice compared to *WT*. However, a closer look at the regenerating areas in muscles from *Hsp70* ^−/−^ mice suggested that myofiber regeneration may still be impaired. We therefore quantified the CSA of regenerating myofibers (only those fibers containing centralized nuclei) and found that these regenerating myofibers were significantly smaller in *Hsp70* ^−/−^ mice compared to *WT* (Figure 9F and 9G). Furthermore, the extracellular tissue surrounding myofibers in regenerating areas of the muscle was quantified and found to be significantly greater in *Hsp70* ^−/−^ mice when compared to *WT* mice (Figure 9H). Therefore, similar to our findings using cardiotoxin injury, these data indicate that myofiber regeneration is also impaired in *Hsp70* ^−/−^ mice following physiological injury induced my muscle reloading. However, since less myofiber regeneration was necessary in muscles of *Hsp70* ^−/−^ mice due to reduced myofiber damage and degradation in the first few days of muscle reloading, overall muscles from *Hsp70* ^−/−^ mice appeared to be closer to morphological recovery than *WT*. Since the average size of all myofibers in the muscle and ultimately the functional properties of the muscle are the most important indicators of muscle recovery following injury we further measured fiber CSA of all myofibers in 10-day reloaded muscles (Figure 9I). No difference was found between the average fiber CSA of myofibers in 10-day reloaded muscles of *WT* and *Hsp70* ^−/−^ mice.

**Figure 10:**
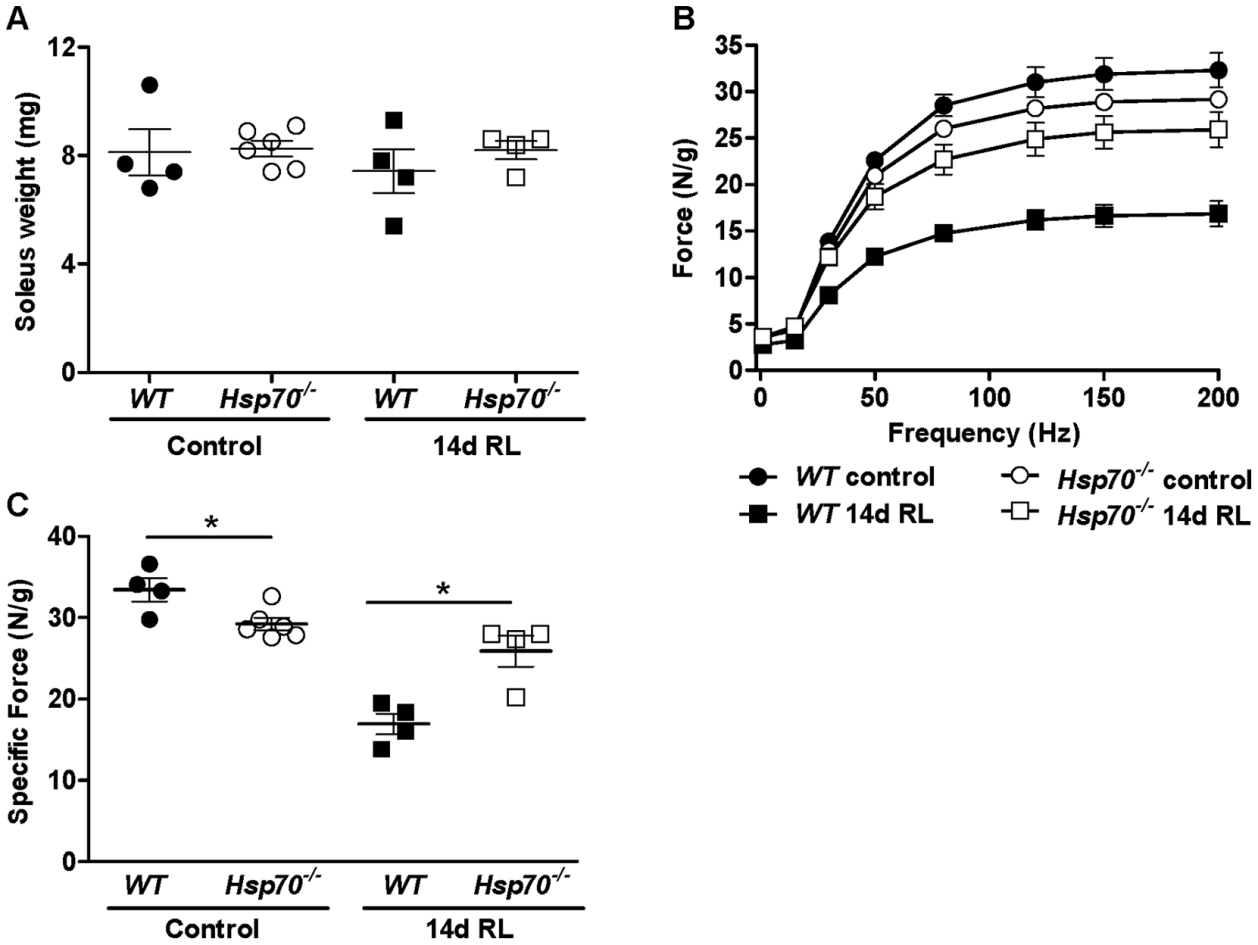
Contractile properties of control and 14-day reloaded skeletal muscles of *WT* and *Hsp70* ^−/−^ mice. *WT* and *Hsp70* ^−/−^ mice were cast-immobilized for 10 days followed by cast-removal and reambulation for 14 days. (A) The muscle weight of control solei and 14-day reloaded solei used in muscle function experiments. (B) The specific force frequency relationship of control solei and 14-day reloaded solei. (C) Maximum specific force of control solei and 14-day reloaded solei. All data represent mean ± SEM, and all data are representative of at least n = 4 mice/group per time point, *P<0.05.

To determine whether differences existed in the functional properties of reloaded muscles of *WT* and *Hsp70* ^−/−^ mice we further performed *in vitro* muscle contractile measurements on soleus muscles isolated from control mice and mice subjected to 10 days of hind limb cast-immobilization followed by 14 days of muscle reloading. Soleus muscle weight is shown in Figure 10A, and demonstrates that the average weight of 14-day reloaded muscles is comparable to control muscles in both *WT* and *Hsp70* ^−/−^ mice. The force frequency relationship normalized to muscle weight is shown in Figure 10B, with maximum specific force further plotted in Figure 10C. Under control conditions, maximal specific force of solei from *Hsp70* ^−/−^ mice was decreased by ∼12% compared to *WT* (*WT*  = 33.44±1.4 N/g; *Hsp70* ^−/−^  = 29.23±0.757 N/g), which demonstrates that under control conditions, Hsp70 is necessary for normal maximum isometric muscle strength. In contrast, in 14-day reloaded solei, the maximal specific force generated by solei of *Hsp70* ^−/−^ mice was 53% greater than reloaded solei from *WT* mice (*WT*  = 16.95±1.26 N/g; *Hsp70* ^−/−^  = 25.89±1.90 N/g). Although muscle weight and the average muscle fiber CSA were not significantly different between reloaded muscles of *WT* and *Hsp70* ^−/−^ mice, reloaded muscles from *Hsp70* ^−/−^ mice showed substantially fewer regenerating fibers. Regenerating myofibers have distinct gene expression profiles from non-regenerating myofibers, which includes their expression of the embryonic and fetal forms of myosin heavy chain. Therefore, it seems plausible that the reduced myofiber damage and subsequent reliance upon muscle fiber regeneration to replace damaged areas of the muscle in reloaded muscles of *Hsp70* ^−*/*−^ mice likely contributed to the enhanced force production of muscles compared to *WT* following the 14 day reloading period.

## Discussion

The current study provides the first evidence that Hsp70 is necessary for normal skeletal muscle fiber size and function under baseline conditions, and is further necessary for normal muscle regeneration and recovery following muscle injury. Importantly, our findings indicate that Hsp70 regulates the regenerative process not only through its intracellular functions in regenerating myofibers, but through acting as a novel regulator of the early inflammatory response to muscle injury.

Twenty-four hours following muscle injury, the increased expression of pro-inflammatory cytokines and markers of immune cell infiltration seen in muscles from *WT* mice were drastically reduced, or even absent in muscles from *Hsp70* ^−/−^ mice. This blunted early inflammatory response in *Hsp70* ^−/−^ mice was associated with significant impairments in muscle regeneration and recovery at later time points. These impairments included smaller regenerating fibers, increased and sustained muscle inflammation, as well as collagen and calcium deposition that remained evident in muscles up to 6 weeks post-injury. Although whole-body *Hsp70* ^−*/*−^ mice were used in this study, through rescue experiments reintroducing Hsp70 specifically into skeletal muscle, we provide strong evidence for a muscle cell autonomous role of Hsp70 in regulating the muscle injury response. Furthermore, through reintroducing Hsp70 into the muscle at two different time points (either 4 days prior to injury or post injury), we provide further evidence that Hsp70 plays an especially critical role in in the first four days following severe injury to promote successful regeneration and recovery. Indeed, while reintroduction of Hsp70 into muscles of *Hsp70* ^−*/*−^ mice at either time point rescued the deficits in regenerating fiber size, reintroduction of Hsp70 into muscles prior to injury also prevented the sustained presence of inflammatory lesions and necrotic muscle fibers seen at later time points. This level of rescue was not similarly provided when Hsp70 was reintroduced into the muscle on the fourth day following injury. Since the first several days following muscle injury are dominated by muscle fiber degeneration, inflammatory cell infiltration and phagocytosis of necrotic cellular debris, our data provide strong evidence that Hsp70 promotes efficient muscle healing following injury through regulating these immune cell-mediated events. The activation and recruitment of immune cells to injured muscle and the removal of necrotic cellular debris via phagocytosis is important for efficient regeneration following severe injury, since disruptions in these events slows muscle regeneration and recovery [31], [33]. Interestingly, Hsp70 was recently shown to enhance the healing process of skin wounds through activating macrophage phagocytic activity and the removal of necrotic cellular debris [29]. It therefore seems plausible that the impaired recruitment of these phagocytic immune cells to injured muscles of *Hsp70* ^−*/*−^ mice may have contributed to the persisting necrotic muscle fibers and slowed rate of muscle recovery. Furthermore, since we were able to reactivate immune cell infiltration into injured muscles of *Hsp70* ^−/−^ mice through direct injection of recombinant Hsp70 protein into the muscle at the time of muscle injury, this provides further evidence that Hsp70 released from damaged muscle may drive the early inflammatory response to injury.

Hsp70 is an ideal candidate in the regulation of the skeletal muscle inflammatory response to injury due to its identified roles in regulating immune cell function when localized to the extracellular environment. Indeed Hsp70 is a known endogenous ligand for immune cell receptors, can stimulate both neutrophil [30], [49] and macrophage [28], [29] activation and chemotaxis, and is sufficient to activate the complement system [50]. Hsp70 may be released into the extracellular environment of injured skeletal muscle tissue from necrotic cells that have lysed, or from viable cells via active secretion to act as a danger signal [51], [52]. Since Hsp70 may be released from cells as either a soluble protein or contained in exosomes [53], future experiments to determine if Hsp70 is passively and/or actively released from injured skeletal muscle tissue and specifically skeletal muscle cells in either of these forms certainly warrants further investigation.

Our findings provide strong evidence that Hsp70 expressed in skeletal muscle is necessary for immune cell recruitment, removal of necrotic cellular debris and efficient muscle regeneration and recovery following muscle injury. However, the majority of these conclusions were derived from data generated using the cardiotoxin model of muscle injury. Cardiotoxin-injection into skeletal muscle is a standardized, highly reproducible model of severe muscle injury that results in necrosis of greater than 90% of myofibers [40]. Therefore, the recruitment of inflammatory cells and the removal of necrotic cellular debris by these phagocytic cells in cardiotoxin-injured muscles may be considerably more important to muscle recovery than in more physiological forms of muscle injury that are less severe. In support of this, studies have shown that immune cell infiltration into muscle can also contribute to secondary muscle fiber damage. This has been demonstrated following muscle reloading injury [54], muscle stretch injury [55] and direct muscle trauma [56]. Therefore, our finding of decreased loss of muscle fiber tissue in 3-day reloaded muscles and increased muscle function in 14-day reloaded muscles of *Hsp70* ^−/−^ mice compared to *WT* mice could more accurately represent the physiological relevance of Hsp70 knockout on the muscle injury response, and could feasibly be explained by decreased infiltration of immune cells in the early days of muscle reloading. However, since we did not measure muscle fiber CSA or function in skeletal muscles of *WT* or *Hsp70* ^−*/*−^ mice in response to immobilization alone, we cannot rule out the possibility that differences in the skeletal muscle response to disuse in these mice may have affected the response to subsequent muscle reloading. Importantly, despite the overall enhanced morphological and functional properties of reloaded muscles from *Hsp70* ^−*/*−^ mice, these mice still showed evidence for impaired muscle fiber regeneration in this physiological model of muscle injury. Indeed, although significantly fewer regenerating fibers were present in 14-day reloaded muscles of *Hsp70* ^−*/*−^ mice compared to *WT*, the average CSA of these fibers was significantly smaller than *WT,* and the extracellular tissue surrounding these regenerating myofibers was increased compared to *WT*. This data is therefore similar to that collected using cardiotoxin-induced muscle injury, and together indicate that Hsp70 is necessary for normal muscle fiber regeneration. Since we found comparable levels in the gene expression of the typical myogenic markers in muscles of *WT* and *Hsp70* ^−*/*−^ mice at time points in which satellite cell activation and early differentiation are near maximum, these data suggest that an impaired ability to increase the myogenic program is not the primary explanation for the regenerative deficits in these mice. However, since muscles from *Hsp70* ^−*/*−^ mice rescued with Hsp70-EGFP showed visual increases in both regenerating myofiber size and the number of centralized nuclei per transfected myofiber, Hsp70 may regulate later stages of the myogenic program to promote myoblast fusion. More detailed analyses into the role of Hsp70 in regulating the different stages of the myogenic program are therefore necessary. Impaired regeneration and recovery in reloaded muscles from *Hsp70* ^−/−^ mice could also be related to the blunted inflammatory response found in these mice, since we know immune cells also secrete factors which support muscle regeneration and recovery [32], [41]. However, given the several known intracellular functions of Hsp70, the failed regrowth of regenerating myofibers in *Hsp70* ^−/−^ mice could be due, at least in part, to impairments in the intracellular processes in which Hsp70 regulates.

Hsp70 is well known to regulate protein quality control. Through its chaperone function, Hsp70 plays a key role in folding newly synthesized proteins, refolding misfolded proteins and transporting irreversibly damaged proteins to the proteasome for degradation to prevent protein aggregation. The removal of damaged cellular proteins and increased synthesis of muscle proteins are undoubtedly critical processes involved in the regrowth and repair of muscle fibers following damage [57], [58]. Therefore, it would not be surprising if the deficits in muscle fiber regeneration and recovery seen in *Hsp70* ^−/−^ mice in this study are also related to impairments in protein quality control due to the absence of intracellular Hsp70. It is well documented that upregulation of intracellular Hsp70 in skeletal muscle protects against muscle damage, enhances regenerating fiber size, promotes functional sparing and preserves myofiber size during atrophy conditions [8], [16], [17], [18], [59]. Perhaps the most striking finding related to Hsp70 overexpression was the recent study published by Gehrig et al., who demonstrated that Hsp70 transgenic overexpression in skeletal muscle and/or pharmacological induction of Hsp70 could improve muscle quality and function in a mouse model of muscular dystrophy. The morphological and physiological benefits provided by Hsp70 to the muscle in each of these studies are likely related, in part, to its role in protein quality control. However, the protective mechanisms of Hsp70 intracellular induction in these studies were also linked to its promotion of calcium homeostasis through regulating the activity of the sarco/endoplasmic reticulum calcium-ATPase (SERCA) complex [19], its antioxidant properties [18] and its repression of the NF-κB signaling pathway [8] which is known to negatively regulate both muscle mass and fiber regeneration [10], [11]. Therefore in the current study the myofiber calcifications, sustained inflammation and deficits in regenerating fiber size seen in muscles lacking Hsp70 may be related to several of these known intracellular functions of Hsp70. Future experiments to tease out the mechanistic and functional differences between extracellular Hsp70 and intracellular Hsp70 on skeletal muscle fiber size the muscle regenerative process are important next steps to fully understand the specific time point and location in which Hsp70 should be targeted to optimally preserve skeletal muscle mass and enhance regenerative capacity.

Importantly, this work provides novel insight into skeletal muscle biology during aging. Hsp70 expression, and the ability to induce Hsp70 expression in skeletal muscle during stress conditions is significantly blunted in aged populations, and is associated with the age-related loss of muscle mass and impaired regenerative capacity [60], [61], [62]. Therefore, our findings that Hsp70 knockout decreases skeletal muscle fiber size and function during baseline conditions and impairs muscle fiber regeneration following injury, provides the first direct link between Hsp70 and these age-related deficits in skeletal muscle plasticity. Modulation of skeletal muscle expression of Hsp70 in aged populations may therefore be an ideal target to promote the maintenance of skeletal muscle mass and function. However, since whole body *Hsp70* ^−*/*−^ mice were used in the current study, the deficits in muscle fiber size under baseline conditions and the impairments in muscle regeneration in these mice could be related to the absence of Hsp70 in any cell type which ultimately influences muscle fiber size and regeneration. Through our skeletal muscle-specific rescue experiments, however, we provide strong evidence that skeletal muscle-derived Hsp70 is indeed necessary for both the normal immune response to injury and muscle fiber regeneration. Nonetheless, future investigations comparing the ability of isolated Hsp70 null myoblasts to proliferate, differentiate, and respond to growth promoting and atrophy promoting signals are important next steps in further establishing the muscle cell autonomous roles of Hsp70 in regulating muscle size and regenerative capacity. Ideally, skeletal muscle-specific *Hsp70* ^−*/*−^ mice would also be used to further establish the muscle cell autonomous roles of Hsp70 in regulating these muscle processes, *in vivo*. However, since such mice are not currently available, the creation of these mice would be of monumental value in furthering our understanding of the biological functions of skeletal muscle-derived Hsp70 in regulating muscle plasticity.

In summary, the findings in the current study demonstrate that Hsp70 is necessary for normal skeletal muscle fiber size and function under baseline conditions and also identify Hsp70 as a novel regulator of the inflammatory response to muscle injury. In addition, this work also demonstrates that Hsp70 is necessary for normal muscle fiber regeneration which we further indicate may be related to the divergent functions of Hsp70 on skeletal muscle plasticity when localized as an intracellular versus extracellular protein. Collectively our findings suggest that combined therapeutics which differentially target extracellular and intracellular Hsp70 could optimally preserve muscle integrity and promote muscle regeneration. These findings are relevant to a wide range of physiological and pathophysiological conditions which lead to increased susceptibility to muscle injury, including modified muscle use, muscular dystrophy and aging.

## Materials and Methods

### Ethics Statement

All animal work was conducted with strict adherence to the guidelines on the recommendations in the Guide for the Care and Use of Laboratory Animals of the National Institute of Health. The protocol was approved by the University of Florida Institutional Animal Care and Use Committee (protocol #201004286). All efforts were made to minimize suffering, and in the event of pain buprenorphine was administered (0.05 mg/kg). At the terminal endpoint of experiments, muscles were surgically removed from mice under isofluorane anesthesia and euthanasia ensured via cervical dislocation.

### Animals and Models of Muscle Injury

Male, *B6.129S7-Hspa1a/Hspa1b^tm^* mice which have a targeted deletion in the coding region of *Hspa1a* (also referred to as *Hsp70.3*) and *Hspa1b* (also referred to as *Hsp70.1*) and hereafter referred to as *Hsp70* ^−/−^ mice, were originally generated at the US Environmental Protection Agency [63] and were purchased from the Mutant Mouse Regional Resource Center (MMRRC) at the University of California, Davis. Their wild-type male counterparts, *B6.129SF2/J* mice (hereafter referred to as *WT* mice) were purchased from Jackson Laboratories (Bar Harbor, ME). All animals were housed in a sterile, pathogen-free, temperature-controlled facility on a normal 12-hr light/dark cycle, and standard diet and water were provided ad libitum.

To induce severe muscle degeneration/regeneration, 100 µl of 10 µM cardiotoxin (Calbiochem) dissolved in PBS was injected into either the right or left tibialis anterior (TA) muscle as described previously [40], of 7–8 week old *WT* or *Hsp70* ^−/−^ mice under isofluorane anesthesia. Muscles were harvested 1, 4, 6, 16, 28 or 42 days post-cardiotoxin injection, as indicated. The uninjured, contralateral TA muscles from each animal served as controls. Physiological injury induced by muscle reloading was induced in 12-week old mice via bilateral hind limb cast-immobilization as described previously [8], with ankles in the plantar-flexed position for 10 days followed by cast-removal and reambulation for either 3 or 10 days prior to tissue harvest. Cast-immobilization and cast-removal were performed under isofluorane anesthesia. Skeletal muscle reloading-injury in the soleus has been demonstrated by numerous lab groups following both hind limb immobilization as performed in the current study [48] as well as following hind limb suspension [42], [64]. Normal weight bearing mice served as the controls for muscle reloading experiments. All experiments were performed in at least n = 3 mice per group, per time point for histological measurements, and n = 4 mice per group, per time point for gene expression analyses and muscle function experiments.

### BrdU Labeling of Proliferating Cells

To measure cellular proliferation in TA muscles in response to cardiotoxin-injury, mice were injected intraperitoneally (IP) daily for 6 days with 50 mg/kg Bromodeoxyuridine (BrdU) dissolved in sterile saline beginning 6 hours prior to cardiotoxin-injury. Injured and contralateral non-injured TA muscles were surgically removed 6 days post-cardiotoxin-injury, embedded in OCT freezing medium and frozen in liquid nitrogen-cooled isopentane prior to storage at −80°C.

### Histology

Skeletal muscles from *WT* and *Hsp70* ^−*/*−^ mice to be used for histology were removed, embedded in OTC and immediately frozen in isopentane cooled in liquid nitrogen prior to storage at −80°C. Prior to sectioning, muscles were equilibrated at −20°C for 1 hour. A microtome cryostat was then used to cut 10-µm-thick serial transverse sections, which were transferred to positively charged glass slides. Sections were allowed to dry for 1 hour at room temperature prior to freezing at −80°C until further processing or stained immediately using H&E to determine morphology. All sections were visualized using a Leica DM5000B microscope (Leica Microsystems Bannockburn, IL) and non-overlapping images of entire muscle cross-sections were captured for 4 sections separated by at least 50 µM per muscle. Each histological analysis was performed on muscles from at least 3 mice per group, per time point.

### Hematoxylin & Eosin (H&E) Staining

H&E staining of muscle cross-sections was performed as described previously [48]. Briefly, slides were brought to room temperature prior to sequential submersions in the following solutions: 100% ethanol for 1 min, 70% ethanol for 1 min, dH_2_0 for 2 min and Gill's Hematoxylin for 2 min. Sections were then washed thoroughly in dH_2_0 followed by sequential submersions in the following solutions: Scott's Solution for 15 seconds, dH_2_0 for 2 seconds, 70% ethanol for 1 minute, Eosin for 2 minutes, 95% ethanol with gentle shaking for 1 minute, 100% ethanol for 30 seconds and Xylene for 3 minutes. Slides were allowed to dry for 30 minutes and then mounted with glass cover-slips using Permount.

### Von Kossa and Trichrome Staining

Slides containing fresh frozen muscle cross-sections were sent to the University of Florida Pathology Core Facility for Masson's Trichrome staining to detect collagen, or Von Kossa staining to detect calcium deposits. The average number of calcifications in entire muscle cross-sections was approximated using ImageJ software from at least 4 sections per muscle, n = 3 mice per group.

### Secondary Immunofluorescence

Sections were fixed in ice-cold acetone for 30 seconds, allowed to dry for 2–3 minutes and blocked in Pierce Superblock (1/20) diluted in 1× PBS for 15 minutes. Sections were then incubated in primary antibody diluted in blocking buffer for 1hr at room temperature in a humid chamber. Primary antibodies for the following proteins were used: embryonic myosin heavy chain (1∶100, Developmental Studies Hybridoma Bank, University of Iowa, Iowa City, IA) and laminin (1∶275, Sigma Aldrich). Sections were washed 3×5 minutes in PBS prior to incubation with the appropriate fluorescently-conjugated secondary antibodies (Molecular Probes, Invitrogen) in blocking solution for 1 hour at room temperature. CD68-AlexaFluor 488-conjugated primary antibody and Ly-6B.2 Alexa Fluor 647-conjuaged primary antibody (1∶175, AbD Serotec), when used, were added during the secondary step. Sections were then washed 3×5 minutes in PBS and mounted with cover-slips using VECTASHIELD® fluorescence mounting medium with or without Dapi (Vector Laboratories, Inc). For muscle cross-sections taken 1 day post-injury, the average number of CD68+ and Ly6B.2+ cells per 20× field was approximated using ImageJ software from at least 4 sections per muscle, n = 3 mice per group. For muscle cross-sections taken 16 days post-injury, the average number of CD68+ cells and/or muscle fibers showing CD68+ staining was approximated in non-overlapping images of entire cross-sections using ImageJ software.

### BrdU Immunostaining

Muscle cross-sections were rehydrated briefly in PBS prior to antigen retrieval with citrate buffer (1.8 mM citric acid and 8.2 mM sodium citrate, pH 6.0) as described previously [65]. Endogenous peroxide activity was inhibited via incubating sections in 0.3% hydrogen peroxide for 5 minutes. Sections were washed in PBS and then incubated in primary antibody (anti-BrdU, 1∶100, Roche Diagnostics, Indianapolis, IN, USA, and anti-laminin, 1∶275, Sigma Aldrich) overnight at 4°C. The following day sections were washed extensively with PBS prior to incubation with secondary antibodies (Alexa Fluor 594 anti-mouse IgG and Alexa Fluor 488 anti-rabbit IgG; Invitrogen) for 1 hour at room temperature. Sections were washed extensively, and then mounted with cover slips using VECTASHIELD® fluorescence mounting medium containing Dapi (Vector Laboratories).

### Quantification of Muscle Regeneration and Fiber CSA

Quantitative analysis of the number and CSA of centrally nucleated fibers (CNF) undergoing regeneration was performed on TA and soleus muscle cross sections stained with H&E (n = 3 mice/group per time point). Four serial cross-sections separated by at least 50 uM were analyzed per muscle. Representative images from non-overlapping fields were visualized and captured for each section using a Leica DM5000B microscope (Leica Microsystems, Bannockburn, IL). Fibers undergoing regeneration were identified as those containing centrally located nuclei. Regenerating fiber cross-sectional area (CSA) and the number of regenerating fibers in entire cross-sections was manually calculated in muscles 16 days post-cardiotoxin injection or following 10 days of muscle reloading, using Leica Application Suite software (version 3.5.0). This software was similarly used to calculate the average CSA of all fibers in serial sections from muscles 28 and 42 days post cardiotoxin-injury and 10 days following muscle reloading.

### Quantification of Extracellular Space

The relative area of extracellular tissue surrounding muscle fibers in muscle cross-sections was approximated using ImageJ software. Images captured from H&E-stained muscle cross-sections were converted to binary images, resulting in eosin/hematoxylin-stained cells and myofibers converted to black and extracellular non-stained areas converted to white. The amount of extracellular space surrounding muscle fibers in was therefore estimated by calculating the percentage of white-coloration per 20× field. Importantly, skeletal muscle cross-sections to be stained with H&E for analyses of morphology were processed immediately following cryosectioning to avoid the introduction of morphological artifacts to muscle cross-sections that may occur in response to freeze-thaw.

### RNA Isolation, cDNA Synthesis and qRT-PCR

Trizol-based RNA isolation from skeletal muscle and generation of cDNA using Ambion's RETROscript first strand synthesis kit (Ambion, Austin, TX, USA) has been detailed previously by our lab group [8]. Prior to qRT-PCR, cDNA was diluted 1/20 in dH_2_0 and used as a template for real-time PCR using a 7300 real-time PCR system and primers purchased from Applied Biosystems (Foster City, CA, USA) based on the following Gene Bank accession numbers: NM_010479.2 (*Hspa1a*), NM_010866.2 (*Myod1*), NM_031189.2 (*Myogenin*), NM_008781.4 (*Pax3*), NM_011039.2 (*Pax7*), NM_001082960.1 (*Itgam/Cd11b/Mac-1*), NM_009853.1 (*Cd68*), NM_010824.2 (*Mpo*), NM_013693.2 (*Tnf*), NM_008337.3 (*Ifng*), NM_008361.3 (*Il1b*), NM_031168.1 (*Il6*), NM_021283.2 (*Il4*), NM_010548.2 (*Il10*) and NM_008355.3 (*Il13*). Quantification of PCR products was performed using a relative standard curve, and the relative quantities of mRNA were then normalized to the levels from uninjured (control) muscles from *WT* mice, and are therefore expressed as fold change. In the event that mRNA transcripts were undetected in *WT* muscles during control conditions, relative mRNA levels are reported in arbitrary units.

### In vivo, Muscle Transfection/Rescue Experiments

The Hsp70-EGFP expression plasmid was created by us, and both Hsp70-EGFP and EGFP have been used by our lab previously [8]. In vivo plasmid DNA injection and electroporation has been described previously by our lab [66]. Muscle transfections into TA muscles of *Hsp70* ^−/−^ mice were performed either 4 days prior to, or 4 days following cardiotoxin-injury.

Low-endotoxin, recombinant human Hsp70 (rHsp70) (Enzo Life Sciences, Farmingdale, NY) was injected into TA muscles of *Hsp70* ^−/−^ mice at the time of muscle injury by dissolving 20ug of rHsp70 into the cardiotoxin-PBS solution immediately prior to cardiotoxin injection. One day following cardiotoxin-injury, muscles were removed and processed for histological or biochemical analyses.

### In vitro muscle contractile properties

The solutions and methods used for studies of soleus muscle isometric function were described in detail previously [67], [68], [69]. Briefly, mice were anesthetized using isofluorane (5%, induction; 3% maintenance), and the soleus muscle excised and immediately placed in buffer solution for dissection. One end of the muscle was tied to a Dual-Mode Muscle Lever System (300C-LR, Aurora Scientific Inc, Aurora, Canada) and the other end onto a secured glass rod using a 4.0 gauge silk suture. Muscles were placed at optimal length (Lo) and allowed 20 min of thermo-equilibration to 32°C. Thereafter, measurements of force-frequency were initiated. In all electrical stimulations a supramaximal current (600–800 mA) and 0.25 ms pulse duration were delivered through a stimulator (701C, Aurora Scientific Inc.), while train duration for isometric contractions was 500 ms. At the end of the protocol, we measured muscle weight to determine specific force (in N/g). All data were recorded and analyzed using commercial software (DMC and DMA, Aurora Scientific).

### Statistics

The Mann-Whitney two-tailed unpaired t-test was used to calculate statistical significance when comparing only 2 groups. Data sets containing more than 2 groups were analyzed using ANOVA with Tukey post-test comparisons. A paired t-test was used to calculate statistical significance when comparing treated and non-treated muscles within animals. All statistical analyses were calculated in GraphPad Prism and a P value less than 0.05 was considered significant.
